# Bacteriophage P22 Capsid as a Pluripotent Nanotechnology Tool

**DOI:** 10.3390/v15020516

**Published:** 2023-02-13

**Authors:** Victor Alejandro Essus, Getúlio Silva e Souza Júnior, Gabriel Henrique Pereira Nunes, Juliana dos Santos Oliveira, Bruna Mafra de Faria, Luciana Ferreira Romão, Juliana Reis Cortines

**Affiliations:** 1 Laboratório de Virologia e Espectrometria de Massas (LAVEM), Departamento de Virologia, Instituto de Microbiologia Paulo de Góes, Universidade Federal do Rio de Janeiro, Rio de Janeiro 21590-902, Brazil; 2 Instituto de Ciências Biomédicas, Universidade Federal do Rio de Janeiro, Av. Carlos Chagas Filho, 373, CCS, Bl. F026, Rio de Janeiro 21941-590, Brazil

**Keywords:** bacteriophage P22, virus-like particles, nanoparticle, protein cage, nanotechnology

## Abstract

The *Salmonella enterica* bacteriophage P22 is one of the most promising models for the development of virus-like particle (VLP) nanocages. It possesses an icosahedral T = 7 capsid, assembled by the combination of two structural proteins: the coat protein (gp5) and the scaffold protein (gp8). The P22 capsid has the remarkable capability of undergoing structural transition into three morphologies with differing diameters and wall-pore sizes. These varied morphologies can be explored for the design of nanoplatforms, such as for the development of cargo internalization strategies. The capsid proteic nature allows for the extensive modification of its structure, enabling the addition of non-native structures to alter the VLP properties or confer them to diverse ends. Various molecules were added to the P22 VLP through genetic, chemical, and other means to both the capsid and the scaffold protein, permitting the encapsulation or the presentation of cargo. This allows the particle to be exploited for numerous purposes—for example, as a nanocarrier, nanoreactor, and vaccine model, among other applications. Therefore, the present review intends to give an overview of the literature on this amazing particle.

## 1. Introduction

Recent years have been marked by a growing interest in the field of nanotechnology, especially its applications in health science. This has been due to the development of new technologies on a nanometric scale, an increase in the demand for alternative or more efficient methods in the treatment of diseases such as cancer, or the need to contain growing threats such as the proliferation of antibiotic-resistant bacteria [1,2,3,4,5,6]. The necessity to develop delivery techniques with a high targeting capacity and an optimized carrying performance of therapeutic agents becomes of the utmost importance to respond to these increasing demands. Nanoparticles present themselves as the ideal tools for this end, thanks to their high versatility and the variety of developed systems [1,2,3,4,5,6].

Nanoparticles can be applied to a great number of functions: because they can act on the healing and regeneration of damaged tissue; as nanometric reaction centers; as the scaffold for the synthesis of organic and inorganic structures; and as carriers for the controlled delivery and release of therapeutic agents, where this last application is one of the most prominent [2,3,4,5,6,7,8]. Because of the small size of these structures that allow for increased tissue permeability and retention, the use of nanometric drug-delivery systems results in a better biodistribution profile and drug pharmacokinetics. This attribute, in combination with target specificity, significantly enhances the bioaccumulation of the carriers in the interior of the mass of tumoral cells and/or the desired tissues [2,8,9]. The targeted delivery of cargo mediated by nanocarriers has some inherent advantages over systemic delivery, such as allowing the cargo to act primarily in its specific reaction site, providing an increased local concentration of the agent, and accessing locations that are normally blocked to conventional treatments [1,3,4,8]. This prevents possible side effects often associated with systemic treatments, such as chemotherapy, while granting enhanced efficiency to them [2,3,4,5]. Better drug distribution can result in a lower dosage necessary for treatments and, consequently, shorten its duration. In turn, it could increase patients’ adherence to treatments with high evasion owing to their decreasing the patient’s life quality, as is the case with the tuberculosis treatment whose abandonment is directly related to the emergence of drug-resistant bacteria [2,4,8].

The vast range of materials that have already been used to design different types of nanoparticles is one of the most prominent virtues of these systems, among which include particles based on lipids, polymers, metals, and proteins. All of them have the potential to promote safe and effective drug transportation through the loading of cargo with therapeutic properties, but their properties significantly vary, where each type has advantages and issues [3,4,5,7,8]. Common problems that may limit the application of nanoparticles include a high production cost and scale-up hindrance, difficult preparation process, restricted administration routes, particle instability, structural heterogeneity, limited biocompatibility, and low target specificity [1,2,3,5,7,10]. However, there is one type of nanoparticle that overcomes some of these limitations: those derived from proteins known to form protein cages. Protein cages are structures that naturally occur in a variety of shapes but are characterized by their self-assembly ability via the interaction of multiple copies of one or more protein subunits, resulting in highly regular hollow nanostructures [6,11,12]. Their application as a standard for the development of nanotechnology is promising thanks to their considerably well-regulated assembly, defined size and structure, biocompatible nature, and high stability [6,11,12]. Their proteic nature confers elevated plasticity that tolerates the extensive manipulation of their structure and has stimulated their ample use as nanoparticles [6,11,12].

Among the developed protein cages, the most prominent are the virus-like particles (VLP) [6,11]. These nanoparticles are derived from viral capsids, being noninfectious homologs of these structures in that they in most cases lack the viral genetic material [5,13,14,15]. VLPs possess nanometric sizes ranging from 10–100 nm, with a highly regular structure capable of self-assembling from multiple copies of protein subunits and, in some cases, from other small molecular components such as RNA. The self-assembly can occur in vivo in the interior of cells of several organisms, such as bacteria or insects, through the heterologous expression of the structural molecule and proteins, promoting the obtention of large quantities of the symmetric and monodisperse population of particles [9,13,14,16,17]. The assembly of these structures occurs through various noncovalent and, consequently, reversible interactions between the subunits. This dynamic aspect of VLPs can be exploited for the directed self-assembly and encapsulation of a considerable repertoire of non-native cargo in its interior. Such an approach has been used in the loading of organic cargo, such as enzymes or peptides, and inorganic cargo, such as polymers or metal atoms [6,13,16,17]. All these elements make the VLPs an extremely promising model for the development of nanoparticles because they retain all the attributes conferred to the viruses through the evolution with and adaptation to their hosts, without the risk of infection [5,14,15,18]. This makes the VLPs a considerably safe and modifiable tool for the transport of cargo through the body.

Among the different VLP models, one of the most promising is the VLP P22 derived from the bacteriophage P22. Over the last decade, the interest in and the research on this particle have been considerably growing, with a great many numbers of papers published demonstrating the ample capacity to modify its structure and the plethora of cargoes that have already been loaded in its interior. This paper aims to perform a nonexhaustive review of the research so far conducted on the VLP P22 as a nanoparticle, paying special attention to its application as a nanocarrier, while also offering a brief record of the history of the research of this exceptional structure and possible routes that the research can take.

## 2. Systematic Review

**Figure 1:**
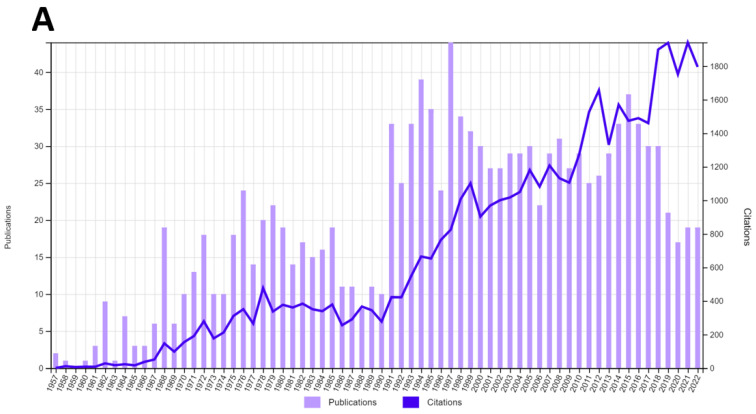

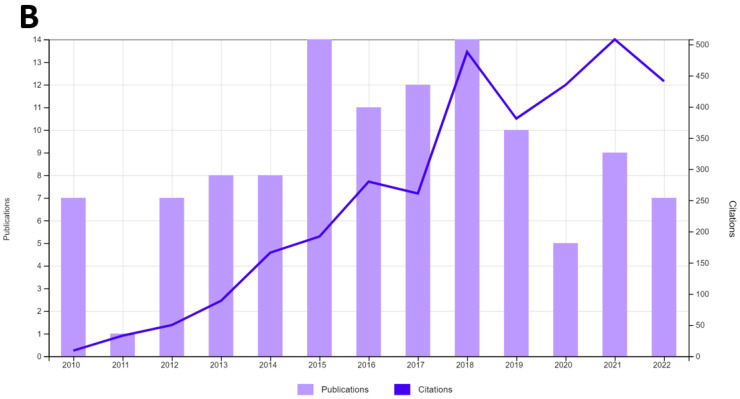
Number of published articles over the years that contain the following terms. (**A**) “bacteriophage p22” OR “phage p22” and (**B**) “VLP P22 OR P22 virus-like particle OR P22 nanoparticle OR P22 nanocarrier”. Research was realized through the Web of Science platform.

The interest in P22 bacteriophage studies has increased since its discovery in 1952. In order to analyze the growth of the phage literature, bibliographic research was conducted by using the Web of Science platform. First, we searched for articles and papers that contained the terms “Bacteriophage P22” or “Phage P22” (Figure 1A), to take a general view of the phage research. From 1957 until December 2022, there were 1299 articles that matched these terms. The number of published papers has considerably grown since 1957, peaking in around 1997, and has remained stable throughout the next 2 decades, showing a slight decline in publication only near the end of the 2010s. On the other hand, citations have steadily grown over time. These consistent publications involving the phage P22 through decades of research paint a clear picture of the growing interest in this virus throughout the years, which has been followed by more profound knowledge of its morphogenesis, structural proteins, and their interactions, as well as its genetic information. To determine the increase in research regarding the application of P22 as a nanoplatform, a search was performed from 2010 up to February 2022, because 2010 was the year when the first paper associating “P22 capsid” to “nanoparticle” was published [19]. The terms used were “VLP P22 OR P22 virus-like particle OR P22 nanoparticle OR P22 nanocarrier”. This search resulted in 113 papers that match those terms (Figure 1B), again with a similar increase the interest in the nanoparticle, highlighting the impact that this technology has had on the P22 research. On the basis of this search, we divided the review according to the most noteworthy applications found in the literature, initially describing the particle structure, followed by the main methods for cargo loading and particle decoration. Subsequentially, we aimed to describe how the P22 VLP has been used to illustrate the current trends in the field. Lastly, we attempted to suggest which path could be explored to expand the uses of this magnificent particle.

## 3. The Structure of the P22 VLP

The P22 bacteriophage is a double-stranded DNA (dsDNA) virus whose natural host is the bacteria *Salmonella enterica sorovar typhimurium*, first described in 1952 by Zinder and Ledeberg [20]. This phage’s morphogenesis relies on the self-assembly scaffolding protein (SP), known as gene product 8 (gp8), and the coat protein (CP, gp5). Its assembly also involves other minor proteins (gp7, gp16, and gp20) and a portal complex ring composed of 12 copies of gp1. SP interacts with CP via the Coulomb interaction to form a competent assembly [21,22,23,24,25,26,27]; these cores interact with other oligomers and protein subunits through the Coulomb interaction [24]; simultaneously, the portal complex is incorporated into the nascent structure mediated by the SP, resulting in an immature capsid, the procapsid (PC). These capsids are built by 60–300 copies of SP and 415 copies of CP, organized in pentamers and hexamers. The resulting structure is icosahedrally shaped (T = 7), with a diameter of ~58 nm; its portal complex is placed on one of the fivefold vertices in an asymmetrical manner with uneven stoichiometry (12:5) to the CP subunits in the vertices; and it possesses ~2.5–3 nm pores in the center of the CP hexamers [13,23,28,29,30,31].

After the procapsid formation, the viral dsDNA is packaged by the minor proteins gp2 and gp3 through the portal complex [22]. The genome packing results in a conformational change in CP monomers and thus in the capsid maturation, followed by a 10–15% diameter expansion and pore shrinkage to 1.3 nm [13,32,33,34]. At the same time, the SP exits through the pores in the center of the CP hexamers. These proteins are not covalently modified, nor do they suffer proteolysis during the assembly, so the SP can play a role in other assembly cycles. Morphogenesis ends with the incorporation of the tail machinery (composed of gp4, gp9, gp10, and gp26) that seals the portal complex opening [22,23,27,34,35,36,37,38]. Unlike the native virus, the P22 VLP is a procapsid-like particle composed solely of CP and SP, lacking the portal complex and tail spike proteins, as will be described below. SP has several functions during capsid formation, including shaping and orienting the PC assembly. Without SP, CP monomers organize in aberrant forms, such as a spiral conformation or malformed capsids [34,39]. It also acts as a catalyst, reducing the subunits concentration needed to start the assembly [35].

**Figure 2:**
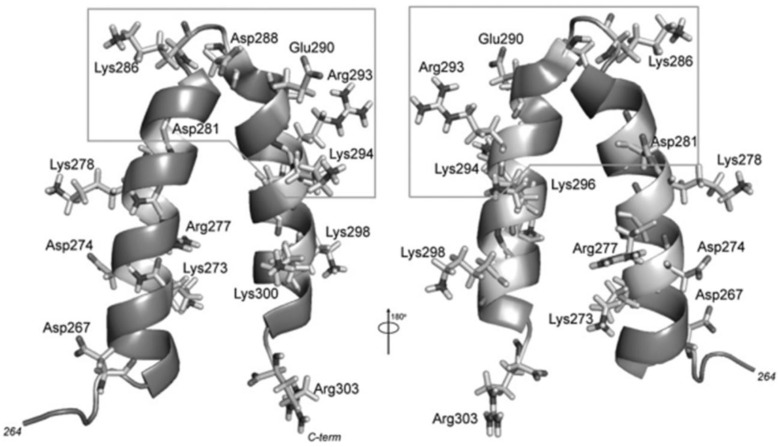
The helix-turn-helix (HTH) domain located on the P22 phage scaffolding protein. The charged residues of this domain are shown in a stick representation. The region highlighted by the box between amino acids 280–294 is the MCBR (adapted from [41]).

The scaffolding protein consists of a long 33.6 kDa molecule with 303 amino acids organized in a mainly helicoidal and flexible structure [27,34,40,41]. The scaffold protein possesses a helix-turn-helix region (HTH) in its C-terminus, comprising amino acids 268–303 (Figure 2), and it is essential for capsid assembly. This region is formed by an 𝛼-helix (H1) linked to another 𝛼-helix (H2) by a five-amino-acid linker (a.a. 284–288), where the first four a.a. forms a β-turn [35,37,41,42]. HTH has an amphipathic nature, where the hydrophobic residues of both helices interact with one another through their side chains to form a hydrophobic core, and SP structural studies have shown this to be more sensitive to amino acid substitutions than the helical surface is. Mutations in the core composing amino acids in H1 impact PC assembly, resulting in a reduction in the number of properly formed particles, where mutations closer to the turn region have a more significant impact [37]. The surface of this region is characterized by a high density of charged residues, presenting five basic amino acids (R293, K294, K296, K298, and K300) in H2, which could explain the SP–CP interaction sensibility to salts [35]. Other charged amino acids may also be involved in assembly and thus also contribute to the formation of the P22 capsid. Inside the C-terminus of SP is the minimal capsid binding region (MCBR) (a.a. 280–296) (Figure 2). MCBR is a positively charged region in which the residues R293 and K296 are the most important for CP binding, which agrees with the fact that changing or removing a considerable part of the SP does not impede the formation of the final capsid structure [43,44]. For the capsid assembly to occur, the MCBR needs to interact with a specific region of the CP protein.

The 47 kDa capsid protein composes the structure of the capsid [21,23,27,45,46]. Each CP subunit is composed of 430 amino acids organized in two main domains, which are connected by a flexible loop region: the N-terminus (a.a. 1–190) and the C-terminus (a.a. 191–430) [44,47,48]. Similar to other dsDNA viruses, e.g., T4 and herpes simplex virus 1 (HSV-1), the CP monomer shares 68% of its structural folding with the HK97 bacteriophage, making it possible to correlate corresponding regions (i.e., structural domains) between the viruses’ monomers [34,49,50]. Moreover, P22 has remarkable features such as not needing an auxiliary protein or a crosslink to stabilize its capsid structure, associated with the CP subunit’s structure [44].

**Figure 3:**
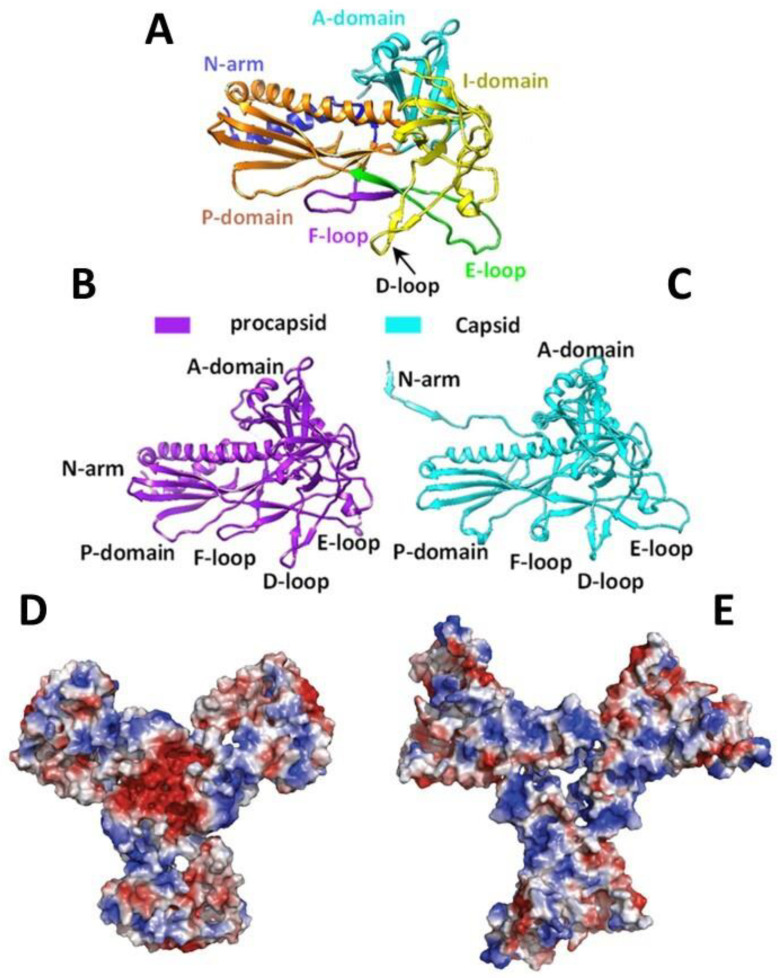
P22 phage coat protein. (**A**) The coat protein monomer is divided into six domains: A-domain, P-domain, N-arm, E-loop, F-loop, and I-domain (D-loop is part of I-domain). (**B**) Immature conformation that is present on PC and (**C**) mature conformation that is present on mature capsid after the dsDNA encapsulation (**D**,**E**) (adapted from [52]). Structure of the “trimer tips”, the meeting point of three hexamers (threefold axis) in (**B**) PC, and (**C**) mature capsid of the three-point axis as viewed from the capsid interior. (**C**,**D**) Structure of the “trimer tips”, the meeting point of three hexamers (threefold axis) in (**C**) PC, and (**D**) mature capsid of the three-point axis as viewed from the capsid interior. The colors represent the surface charge densities of the CP, with red regions representing the negatively charged residues, blue regions representing the positively charged residues, and white regions representing the neutral residues (adapted from [24]).

The CP monomer is divided into domains named according to their position on the capsomer (Figure 3A–C). A-domain (a.a. 128–221, 346–357, and 417–430) surrounds the center of the oligomer and has a flexible loop exposed to solvents. It is a region susceptible to protease action in the monomer and the PC, but it becomes more resistant after capsid maturation [43,48,51,52]. The flexibility of the A-domain allows the manipulation of this region with no repercussions to PC assembly or capsid integrity [43]. One example is residue T183, which is in an axial position on the capsomer, as seen in structural models of P22, making it an interesting target for mutations, especially because it was shown that T183 modifications did not affect capsid assembly [51]. The P-domain (a.a. 31–33, 79–127, and 358–416) is positioned at the extremity of the protein and forms an important interaction with the N-terminal arm (N-arm) in (a.a. 2–30) that stabilizes the capsid after expansion [44,52]. The E-loop (a.a. 51–78) is a hinge-shaped region, which is a site of crosslinks in HK97 but is shortened on the P22 [34,52]. It forms salt bridges with the P-domain of the neighbor subunit, playing a role in capsid stabilization; however, amino acid substitutions in the E-loop apparently did not affect the capsid, according to [50]. The I-domain (a.a. 222–345) is inserted into the A-domain and is absent in HK97 [34,44,50,52]. This domain has a D-loop region forming essential ionic interactions over the local and icosahedral twofold axis that contributes to capsid stabilization. The absence of these interactions results in aberrant and tubular particles [50]. New salt bridges also appear between the N-arm and the F-loop (a.a. 34–50) in the mature capsid [52]. The low energy of the CP subunits interactions makes the structure flexible, to the point of allowing the substitution of monomers in the PC particle with others in a solution. This flexibility can be related to the expansion capacity or capsid integrity given that the dissociation of subunits allows for a thermodynamic selection ensuring that only the correctly structured subunits are added to the particle [53].

It is believed that SP–CP binding occurs between MCBR of the C-terminus of SP (a.a. 280–296) and the N-arm of CP [24,25,35,37,41]. The N-arm is situated in a region called “trimer tips” (Figure 3D,E), which is the meeting point of three hexamers (a threefold axis) in the assembled PC and is negatively charged. The interaction between these two proteins is mainly electrostatic, where the CP D14 residue is primarily responsible for SP binding [24,25,35,37]. Because of the negatively charged nature of DNA, it is believed that the packaging of this molecule repels the negative regions of CP, forcing a conformational change that results in N-arm reorganization from an α-helix to an extended loop and triggering the capsid expansion [13,24,34,35,44,52,54]. The conformational changes of CP initially occur on the A-domain, the P-loop, and the N-terminus, and these last two are the main ones responsible for capsid stabilization during maturation [24,44]. During expansion, the N-arm’s extended loop interacts with the P-loop, closing the distance between the regions and promoting contact between two parts of the subunit, which contributes to increasing the stability of the expanded capsid [34,44,52,54]. A movement occurs on the A-domain (Figure 3C) that projects it toward the center of the hexamer, shrinking the hexamer’s pore and simultaneously increasing intersubunit interaction because the A-domain becomes more prominent, thus granting more resistance to the structure [34,48,52,54]. Thanks to these transformations, the maturation is considered an irreversible process that occurs as a result of the big energetic investment needed to overcome the PC stability, which comes from the ATP-driven dsDNA packaging during morphogenesis [48,53]. The expansion fixes the subunits in symmetry axis and locks them in specific positions, increasing the stability of the capsid, and probably represents a way of balancing the need for flexibility during the assembly and the necessary rigidity for protection during infection [34,53]. This stabilization seems to be essential to P22 as PCs are fragile enough to slowly disassemble at a low concentration of monomers [53]. Another consequence of the N-arm reorganization is the inversion of the charges in this region from negative to positive. Consequently, the binding sites of the scaffolding protein are blocked, resulting in SP disassociation from the capsid and its exit through the pores of hexamers on the A-domain [13,24,34,44].

The exit of SP can also be induced in vitro with no change in capsid morphology. In the PC, it remains attached to the internal surface of the particle, but it can be removed by using low concentrations of chaotropic agents such as urea (0.5 M) or guanidine chloride (GuHCl). The removal of SP results in an empty shell (ES) structure, composed solely of capsid proteins [13]. In addition to that, an increase in ionic strength or temperature also accelerates SP exit [13,25]. This process is reversible, in that the SP can reenter and rebind to empty capsid after the removal of the chaotropic agent and the incubation of ES in a medium with the presence of SP, and if there is an excessive concentration of the protein, it can result in “stuffed shells” [13,27,37]. The P22 capsid assembly can also be promoted in vitro by using only copies of the CP and SP, as it requires only the SP–CP binding to occur, forming the PC-like particle that is used as a VLP [27,34,55].

The VLP particle differs from the bacteriophage in that it possesses 420 copies of CP, instead of 415 copies, and possesses 12 pentamers, having an additional pentamer where the portal complex would be [13,16,17,25]. Like other VLPs, the P22 particles can be obtained by protein heterologous expression, using *Escherichia coli* cells, a relatively simple and well-known method. As mentioned earlier, a large segment of the SP sequence can be changed, and its N-terminus can be considerably truncated with no major consequences to capsid assembly or structure. Previous works have exploited this aspect of the VLP for the in vivo packaging of cargo utilizing truncated variants of SP composed of amino acids 141–303 and 238–303 [16,17,56]. The truncated SP has different properties and effects on the capsid. Its isoelectric point is higher than the wild-type SP (SPwt) one, making it more cationic in neutral pH, thus changing its interaction with the capsid and increasing SP–CP binding affinity. This promotes nonspecific interactions and the retention of the SP inside the capsid [13]. The use of this truncated variant also grants a better utilization of the VLP’s internal space, allowing the packaging of a higher number of protein fusions in the capsid [56]. This way, exogenous proteins can be genetically fused to SP [13,16,57], and other molecules can be encapsulated during the assembly or through the pores after the particle has formed [9]. The capsid protein also might be altered, which enables the tuning of the VLP into a suitable tool for a specific purpose. Helix 1, comprising a.a. 97–130 and situated on the P-domain, is oriented toward the interior of the capsid, and its mutations did not affect the capsid structure, making it a good site for internal modifications [19]. Several residues were already substituted to be used as binding sites for the incorporation of exogenous structures by chemical modifications, e.g., peptides and drugs [9,12,19,56]. Both cargo-loading strategies and particle structural modifications will be explored later.

**Figure 4:**
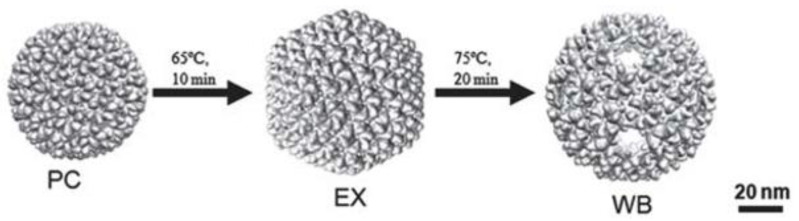
External view of the three P22 VLP morphologies and the process of transformation. Procapsid morphology (PC), 58 nm size and 2.5 nm pores; expanded shell morphology (EX), 65 nm size and 1.3 nm pores; and wiffleball morphology (WB), 65 nm size and 10 nm pores (adapted from [59]).

The P22 VLP versatility comes from protein tolerance toward modification and its multiple capsid morphologies (Figure 4). The capsid maturation process can be reproduced in vitro by heating the particle to temperatures between 65–70 °C for 10–15 min without the need of dsDNA packaging, resulting in the expanded shell (EX) morphology, a structure that is similar to the mature phage’s capsid [16,34,58,59]. In this transition, the internal volume expands by 35%, and the pores are reduced [60]. The further heating of the particle at 75 °C for 20 min causes the release of the 12 pentamers and the obtention of the wiffleball (WB) morphology, a rounder structure marked by the presence of bigger pores of around 10 nm at the vertices where the pentamers were. This structure has the same volume and CP conformation as the EX particle [9,13]. This way, the P22 VLP has three morphologies with distinct properties that can be exploited for multiple ends, such as cargo loading [9,12], particle decoration [61,62], or biophysical studies [54]. These properties will be explained in further details in the topics below. The size and porosity of the capsid and CP conformation depend on VLP morphology. The transformation of PC to EX increases the particle internal volume and stability, reduces pores size, and changes its electrostatic properties [13,25,60]. On the EX-to-WB transition, the exit of pentamers increases particle porosity, expanding the access of substrates to the interior of the particle or the exit of cargo, allowing the insertion of external structures into the particle [9,12].

## 4. Cargo Loading

One of the main focuses of P22 VLP’s application as a nanoparticle is its use as a nanocarrier for targeted delivery. Although this application has already been previously established for other VLPs, Kang et al. were the first to propose the use of the bacteriophage P22 capsid as a model for nanoplatform development [19,43]. The P22 possesses a larger capsid size compared with other spherical viruses used as VLP models, which allows it to encapsulate larger molecules or volumes of cargo [19,59]. It is also notable thanks to its multiple conformations. This structural transformation capacity makes it a versatile tool thanks to changing not only its morphological aspects but also its chemical properties [13,19]. The transformation rearranges the disposition of the amino acid residues thanks to its modifying the architecture of the particle’s subunits, consequently changing the chemical reactivity of certain regions in the capsid, as previously mentioned [19,24]. Such changes in the structural layout can be exploited for the interaction between specific structures, such as cargo molecules. Furthermore, the range of possible chemical interactions can be expanded by replacing native residues with those with properties better suited to the desired end [12,19].

Adding sulfhydryl reactive groups on the VLP interior for postexpression cargo encapsulation was the approach used Kang et al.’s first paper, where cysteine residues were introduced at specific sites in the interior surface, and their reactivity was tested by crosslinking them with maleimide-containing molecules [19]. The substitutions were performed on residues Val 119, Lys 110, and Lys 118, present in helix 1 of the coat protein (CP). Subsequently, the scaffold protein (SP) was removed and the inserted residues’ reactivity was evaluated by testing the residues’ capability of covalently binding with the maleimide-PEO2-biotin (MPB) in the particles’ empty shell (ES) and wiffleball (WB) morphologies. The resulting MPB binding was confirmed by changes in mass, as observed through mass spectrometry analysis. While the V119C mutation did not appear to bind to MPB, both the K110C and K118C mutants showed an increase in mass that varied depending on the particle morphology. The K110C presented a mass difference relative to the presence of MPB in the ES procapsid morphology, but such a change was not seen in the WB form, indicating that the residue became inaccessible owing to the transformation from a procapsid to a WB. On the other hand, 35% of the K118C residues in the procapsid form could bind MPB, and all of them could after the WB transformation, suggesting that the inserted cysteine residue becomes completely exposed. These results illustrate that depending on the particle morphology, different residues are accessible for reactions. Subsequently, they evaluated the potential of the WB particles to internalize and harbor cargo molecules by using streptavidin conjugated to fluorescein (F-StAv) to react with the biotin terminals of MPB. Through SDS-PAGE, it was observed that K118C MPB-labeled WB was loaded with F-StAv in around 13 molecules per particle, which is approximately the number of holes present in its morphology. It was theorized that F-StAv molecules bound themselves to the residues in the pentameric holes not fully entering the particle but preventing the entrance of other molecules. Another point considered was that the StAv molecule was too large to pass through the pentameric holes.

**Figure 5:**
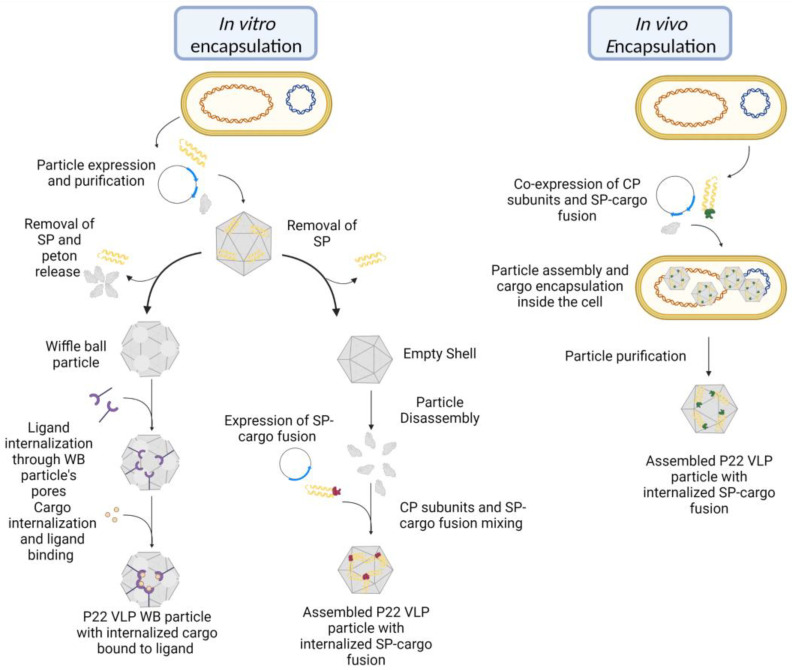
Scheme of different strategies for cargo loading into the P22 VLP. The scheme shows common methods of cargo loading into the P22 VLP, either in vitro (after particle expression and purification) or in vivo (during particle expression inside the cell). In general, the in vitro internalization of cargo using the WB morphology is performed by an initial modification of the particle’s internal surface with a ligand molecule that will then bind to the desired cargo molecule [9,12,63]. Another method of in vitro cargo loading consists of disassembling ES particles to obtain CP subunits and mixing them with a SP–cargo fusion, promoting the assembly of P22 VLP and cargo internalization [13,65,66]. The in vivo encapsulation method consists of the coexpression of CP subunits with SP–cargo fusions, resulting in P22 VLP assembly and cargo loading inside the cell [16]. The figure was created at biorender.com.

This first study illustrated the capability of exploiting engineered mutations for the specific binding of in the P22 VLP interior, the binding of target molecules, and the possible hindrances associated with its entrance in the particle interior [19]. Although the entrance of cargo through the WB particle’s pore was not observed, it would be proven possible and exploited for the in vitro internalization of cargo in subsequent studies (Figure 5) [9,12,63]. The incorporation of cargo postexpression by using the pores present in the different particle morphologies would become a method of cargo internalization, especially for small molecules [64]. However, one of the most frequent methods of encapsidation (which uses the SP protein for in vivo cargo internalization) was established in another study [16], as described below.

The first work to show the capability of the in vivo encapsulation of cargo using the VLP P22 was conducted by O’neil et al. (Figure 5) [16]. In their research, the authors fused the fluorescent proteins mCherry or enhanced green fluorescent protein (EGFP) to the N-terminus of a truncated variant of the SP (141–303). This method yielded 150 mg of VLP per liter of medium where the particles had a radius of 22–25 nm, similar to what was seen in particles that contained solely the truncated SP. Although no significant difference in size was noted between loaded and empty particles, liquid chromatography and mass spectrometry analysis revealed a considerable increase in mass on the former, thus confirming the presence of cargo in its interior. The presence of cargo did not affect the VLP’s capacity to undergo the conformational changes characteristic of the capsid. The EX form was obtained after heating at 65 °C, but the SP remained trapped because the fused cargo was unable to pass through the hexameric pores. However, that was not the case with the WB morphology, which can also be obtained upon heating the particle at 75 °C, in which the SP–cargo fusion was able to exit the capsid through the characteristic 10 nm pores. The cargo was also retained after the particle was treated with heat in order to expand the particle, and the subsequent thrombin cleavage of the fluorescent protein fused to the SP. This thrombin treatment resulted in the SP exit while the cargo remained trapped, representing an efficient way to separate cargo from the SP [16]. Through this study, one of the most common methods of cargo encapsulation in P22 VLPs was established. Among the advantages of SP-mediated cargo loading is the rescue of recombinant proteins that tend to aggregate after expression, thus hindering their application. It was reported that by fusing with the SP, it was possible to the encapsulate the *Pyrococcus furiosus* α-galactosidase (GalA) and the hemagglutinin head (HAhead) region from influenza, both proteins reported to aggregate when in a solution [46]. One drawback of in vivo loading is the inability to regulate the amount of cargo loaded [65,66]. However, one reported solution is to separately express the SP-fused cargo and internalize the cargo in vitro by mixing it with disassembled monomers of CP (Figure 5), increasing control over the available cargo for internalization [13,65,66].

In a subsequent study, it was possible to simultaneously encapsulate two cargo proteins by using this SP-mediated approach [67]. A triple protein fusion consisting of the truncated SP, green fluorescent protein (GFP), and mCherry was developed and expressed, leading to the correct assembly of the P22 VLP particle and the polyprotein encapsulation in vivo. The particles formed were highly homogenous and indistinguishable from WT P22 VLPs while also retaining their structural transformation potential. Furthermore, the internalized proteins retained their characteristic emission and absorption properties, occupying around 24% of the available space in the interior. Therefore, it was possible to successfully obtain a catenated multiprotein loading in a stoichiometrically balanced fashion without affecting particle synthesis. They were also able to analyze the impact of protein confinement in the restricted space of the VLP interior by introducing glycine sequences of varying lengths (6, 12, and 18 Gly) that conferred a certain flexibility to the polyprotein and by analyzing the GFP and mCherry interaction through the Forster resonance energy transfer (FRET). Both proteins are a FRET pair, and under permissible conditions, GFP acts as a donor allowing mCherry to exhibit fluorescence even when the latter is not excited. Both the catenated and glycine spaced proteins were probed when in the PC or EX particle, as well as in a free state. It was observed that when in the PC particle interior, where the molecular crowding was more intense and the proximity of the fluorescent proteins was closer, the FRET value was higher [67]. According to such results, the influence of molecular crowding in the particle’s interior becomes clear. Such factors are of great importance in the development of VLP systems, especially nanoreactors, as will be presented in later parts of the review, and this is a topic of constant study [25,66,68,69,70,71,72,73].

Cargo can have remarkable impacts on the physical properties of the capsid, as seen in a study by Llauró et al. [25]. In it, the effects of cargo in the VLP P22 structure were analyzed through atomic force microscopy (AFM) and three-dimensional cryoelectron microscopy using SP fused to EGFP and a CelB enzyme, a tetrameric β-glucosidase, loaded in vivo. When comparing the rigidity of the native capsid with one loaded with the proteins of interest, an increase was observed in the latter, suggesting that the presence of cargo was responsible for the change. Differences in density in the interior of both loaded and empty VLPs revealed that the majority of the cargo was located in a layer beneath the capsid wall, indicating that the VLP stiffening was a result of structural reinforcement conferred by the encapsulated proteins. The interaction with the capsid structure also differs depending on the cargo, consequently affecting its stability in different ways. Although both EGFP and CelB had a similar influence on the particle rigidity, their effect on its brittleness varied between them when compared with the empty VLP. The interactions between SP fused to EGFP and the internal surface of the capsid were similar to that of SP in the native capsid, where its C-terminus fit in specific points in the CP hexamer while this interaction was preserved. On the other hand, SP fused to CelB showed an unorganized distribution in the same region, where the connections mediated by the SP were irregular and less defined. This indicates that fewer SP–CP connections are made in CelB than in EGFP, possibly thanks to the CelB’s tendency to interact with and form tetramers while EGFP remains a monomer. It was suggested that this oligomerization of CelB would tighten the connections made by SP, inducing geometrical restrictions that would restrict the mobility of CelB subunits, making the particle more fragile than the EGFP-loaded VLP. The VLP morphology also affects the interaction with the cargo, as observed when the particle was expanded. Although an increase in rigidity was also observed for the loaded EX VLP P22, its brittleness was not affected by the presence of cargo, in neither CelB nor EGFP, when compared with the PC morphology. That is possibly due to the conformational changes that occur during expansion, in which the SP–CP binding sites are blocked, resulting in the detachment of SP from the capsid wall and its subsequent release. However, when an SP is fused to cargo molecules, it is unable to leave the capsid and is retained in suspension inside the VLP. This dissociation of the SP from the particle interior would also explain the lack of influence of CelB on its brittleness in that it no longer poses a geometrical hindrance. The increase in particle rigidity is theorized to have been caused by osmotic pressure generated by the difference in concentration between the VLP interior and the surrounding environment because it could not have been due to structural reinforcement. Electrostatic repulsion between the cargo molecules and/or the capsid wall could also contribute to the increase in pressure [60]. Such aspects are important to consider in the design of nanoconstructs, especially when factoring in particle stability and the preservation of cargo integrity.

Another important aspect associated with the interaction between cargo and particle is the capability of the former to exit its interior. For the development of an efficient delivery system, an optimal cargo release strategy must be utilized. Thus, the evaluation of the cargo’s effect and influence on both the SP and the whole particle becomes imperative. The elucidation of the mechanisms that act in this dynamic allow the development of strategies that promote an enhanced release of cargo maximizing the potential of VLP P22 as a nanocarrier. A study evaluated the cargo exit behavior over time with different traits and under different conditions, such as VLP P22 morphology, encapsulated molecule size, varying temperatures and ionic strengths, and SP length [13]. This study utilized GFP and CellB as a cargo molecule, the first because it passed through the WB 10 nm pores and the latter because it had similar proportions to the pores. It was the first to explore the release profile from the PC morphology. To propitiate SP exit, the particles were treated with varying concentrations of GuHCl, and the release of the SP–cargo fusion and the native protein were compared. While the native scaffold protein was successfully removed, both cargoes were unable to leave PC particles when treated with 0.5 M of the chaotropic agent, suggesting that cargo retention was due solely to size limitations and not due to interactions between the SP and the capsid surface. Because the SP folds onto itself when in suspension [74,75], it is possible that cargo fusion can increase the size of the folded protein, making it larger than the 2.5 nm hexameric pore. Even in the absence of heat or chemical treatment, the SP naturally leaves the capsid over time, but a gradient between the ionic strength inside and outside the particle can hasten the process, as it was observed when the VLP was submitted to varying concentrations of salt. In the highest concentrations of salt, the release was immediately faster than the treatment with lower concentrations, supporting the hypothesis that an increase in ionic strength accelerates SP exit, probably thanks to its interfering with electrostatic interactions with the capsid. The length of the SP also affects retention, where truncated variants are retained even when the particle is treated with different concentrations of salt. They are also present in greater numbers than their native counterparts when the particle is assembled in vitro, surpassing even the specific binding sites at the internal capsid surface. That is probably because the deletion of its first 141 residues significantly increases the protein charge at a neutral pH, allowing for nonspecific binding to the negatively charged capsid interior. It was also proposed that the retention was due to the oligomerization of the truncated protein, making it too large to pass through the particle pores. In order to evaluate whether this retention was a result of binding in putative sites or because of an unspecific interaction with the capsid, the particle was expanded through heat treatment because expansion blocks the binding sites, but the particle’s negative charge remained the same. The truncated proteins were retained even after particle expansion, probably because the shrinking of the hexameric pores may have restrained the SP, given that it is unknown whether the native protein is able to pass through them. It is not established whether its exit occurs before or after the capsid expansion. Therefore, if the expansion can happen without releasing the truncated protein, it would explain its presence in the EX particle. However, even when the PC was expanded to the WB morphology, there was still a significant quantity of SP present in the particle interior. Although a change in the molecular weight confirmed the release of the 12 pentons, the retention of a fraction of the truncated protein revealed that it was not only a physical impediment that was keeping them inside the VLP, indicating that other properties act on this interaction. Therefore, the retention of SP variants on the VLP interior is dependent on the particle morphology, which determines whether the putative binding sites are accessible and whether the capsid pore size is large enough to allow cargo passage through the capsid wall [13]. Other studies have developed different strategies for the release of cargo, such as exploiting the environmental pH to induce disassociation from the VLP structure and exit [9,12] or the programmed particle disassembly [76,77]. The vast array of methods for the internalization of cargo makes the varied types of molecules possible, which in turn makes the equally astounding number of applications possible, some of which are summarized in Table 1. Some applications include the delivery of therapeutic cargo [9,12], its use as a vehicle for the transport of contrasting agents [78], the use of the particle interior as a nanoreactor [69], and the carrying of immunogenic cargo for the activation of the immune system [46,79]. The potential of P22 VLP as a nanotool only increases when we remember that there is still another surface to be exploited, in addition to its inner one.

## 5. Modification and Decoration of the External Surface of P22 VLP

**Figure 6:**
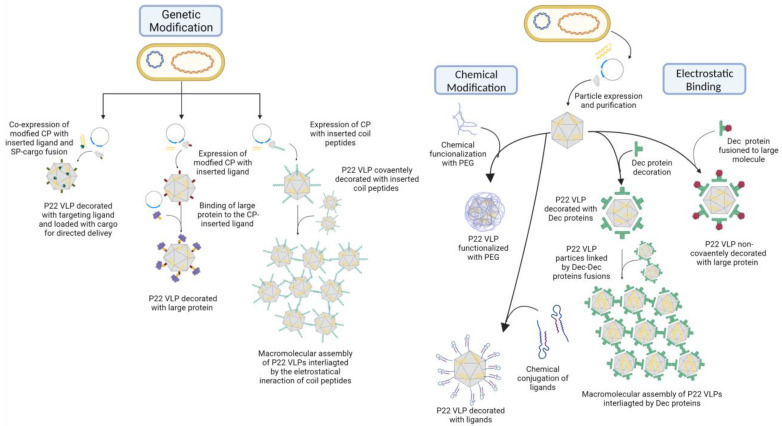
Scheme of different methods for modifying the P22 VLP exterior and particle decoration. On the left, some of the types of structures that were used to genetically decorate the P22 VLP are illustrated, this method consisting of the insertion of a non-native peptide into CPs’ primary sequence. Inserted structures include targeting ligands for the targeted delivery of internalized cargo [9,12,56]; ligand sequences for the annexation of large molecules for molecular presentation or to confer target specificity [12,17,100]; and coil peptides for the development of macromolecular assemblies with P22 VLP [81,87,97]. On the right, the methods for the postexpression modification of VLP particles’ exterior, examples of which include the chemical modification of the particle [9,69,71,85,98] and the electrostatic annexation of foreign structures [60,61,62,92,102]. Chemical modifications include those with polymers such as polyethylene glycol (PEG) [69,71,98] or the insertion of targeting ligands [9,85]. On the other hand, the use of the electrostatic binding of structures can be explored for the annexation of the Dec protein, allowing the incorporation of large molecules for molecular presentation or to confer target specificity [61,62], as well as the development of macromolecular structures [60,102].

In addition to modifying its interior, the plasticity of the P22 capsid allows for extensive modifications to its exterior surface, allowing its use for multiple ends. Similar to what was previously mentioned for other VLPs, VLP P22 can be decorated with a remarkable variety of structures through an equally diverse number of approaches of chemical crosslinking or genetic alteration to the capsid structure [43,51,56,81]. Through these methods, structures such as cell-penetrating peptides (CPPs) [56], ligands [12,62,69], polymers [71], antigens [100,101], large proteins, DNAs, and even nanoparticles. This allows the VLP to be used for molecular display [43,51,81], the presentation of antigens [100,101], targeted delivery [9,12], and the development of complex supramolecular structures [87,90,102]. Figure 6 lists some of the most common methods for particle modification. Modifications on the structure can also be used to develop new strategies to bypass issues commonly encountered in development, such as the delivery of cargo. The incorporation of activated 5-Norbornene-2-carboxylic acid into the exterior of the P22 VLP for the programmed release of cargo under physiological pH has already been reported [76,77].

Among the different types of architecture manipulation, the genetic approaches are particularly preferable thanks to their requiring few steps and their resulting in a higher homogeneity in the particle population. The mutations are carried out in regions that do not harm the VLP structure and simultaneously offer some advantage to its application [51,81]. The C-terminus of the coat protein (CP) subunits tend to be the focus of the incorporation of cargo thanks to its being turned toward the exterior environment and containing the outermost region of the particle. This region is also extremely tolerant to modification, allowing the manipulation of the particle structure without affecting its assembly [51,81]. These aspects make it an ideal site for the annexation of heterologous structures such as antigens because it makes it more easily accessible.

One of the earliest studies to modify the external surface of P22 VLP used the threonine at position 182 (T182) as a focus for mutation [43]. This residue is positioned on a flexible loop of the CP monomer located in the exterior of the assembled capsid and was used to test the capacity for the insertion of structures. They inserted a cysteine residue to serve as base for the specific binding with a thiol-reactive fluorescent marker, MIANS (2-(4′-maleimidylanilino) naphthalene-6-sulfonic acid). After MIANS establishes a covalent bond, its fluorescence heightens, which allows it to be used to probe the interaction with the inserted cysteine residue. To this end, the PC particle was incubated with MIANS to promote binding. Through the measuring of the absorption and fluorescent emission, it was possible to determine the degree of the particle decoration by observing that 345 MIANS molecules were bound per particle, equivalent to about 82% of the available sites. In the same study, a sequence of six histidine molecules (6X-His-tag) was inserted into the T182 position to test whether an external sequence would interfere with particle assembly and whether it could be interacted with. Not only did the particle assemble without deformity, but the histidine sequence was also proven to be accessible to interaction as it bound itself to histidine-specific antibodies and was labeled with Ni-conjugated nanogold [43]. A similar result was observed in another study that focused on investigating the use of the C-terminus for the insertion of non-native peptide sequences [81]. Different P22 VLPs containing or not a 6X-His-tag were applied in a Ni chelate affinity column, and only the one presenting the marker on its C-terminus was retained, which was further confirmed by a sandwich ELISA assay with His-specific antibodies. This study also tested the insertion of a single cysteine residue (431C) and two coiled coil peptides to the region, neither compromising particle assembly or structure [81]. The cysteine residue was susceptible to fluorescein-5-maleimide (F5M) labeling, and it was proven capable of forming disulfide binding between capsids, confirming its presence in the capsid exterior and its accessibility. To further explore possible interactions between particles, they studied the insertion of coiled coil peptides that consist of heptad repeats of amino acids that generate superhelical structure motifs when mixed. Either of the two sequences consisting of 23 residues were inserted in the CP C-terminus, the positively charged K-coil (+TR(VAALKEK)_3_) α-helix, or the negatively charged E-coil (+TS- (VAALEKE)_3_) α-helix, because when mixed together, these peptides form a heterodimer. It was observed that particles containing one of the peptides interacted with particles containing the other almost immediately after mixing, as seen by the increase in light scattering observed by UV spectroscopy, indicating the formation of a larger structure [81]. These results prove the ability to both induce genetic mutations for chemical modifications to the particle exterior and use them to insert non-native structures, aspects that would be extensively explored in subsequent studies [12,56].

One of the most used approaches for such a type of modification is the chemical conjugation of the desired structure to the particle, using the native capsid residues or those that were genetically inserted, allowing for a wide variety of reactions to be utilized for conjugation. One example is the work carried out by Tapia-Moreno et al. [71] that modified the external surface of the VLP withpolyethylene glycol (PEG) heterofunctionalized with folic acid (FA) and N-hydroxysuccinimide (NHS). The NHS moieties of the polymer were covalently attached to the exposed free amino groups of the VLPs by using the succinimide-activated esters. The main goal of pegylation is to mitigate immune responses. Additionally, it increases the half-life and circulation times of the molecules and reduces nonspecific binding to nontargeted areas. The pegylation was also utilized in other materials that have been approved by regulatory authorities and are clinically used with success. Many types of tumor cells are known to often overexpress folate receptors; this way, FA can be a good molecule to recognize the tumor cells, and together with PEG, they can confer great target specificity to the VLP. This was experimentally shown by using cytochrome P450 (CYP), an enzyme frequently used in enzyme prodrug therapy (EPT), as cargo. The results indicated ligand-mediated cell internalization, once the uptake of functionalized VLPs with PEG-FA into the tumor cells was higher than that of the untreated ones, indicated by the catalytic activity inside the cell. These results suggest that the PEG-FA improves VLP entry and confers specificity to tumor cells [71].

Like Tapia-Moreno et al. [71], Chauhan et al. [69] also performed the EPT with CYP, but they combined it with photodynamic therapy (PDT) by adding a photocatalyst, protoporphyrin IX (PpIX), in the external region of the capsid. The carboxylic groups of PpIX were activated to succinimidyl ester using 1-ethyl-3-(3-dimethylaminopropyl)-carbodiimide (EDC) and NHS. Therefore, the functionalized PpIX was conjugated directly to the surface-exposed amine groups of biocatalytic P22 through a carbodiimide reaction. In presence of light, the PpIX produces reactive oxygen species (ROS), increasing the superoxide dismutase activity (SOD), which transforms a O^2-^ radical into hydrogen peroxide (H_2_O_2_), which intensifies the bioactivation of tamoxifen via a H_2_O_2_-driven CYP-oxyfunctionalization chemistry. As it is known that approximately 80% of breast cancer cases are estrogen receptor positive (ER+); an estrogen derivative, 17α-ethynylestradiol (EE2) was chosen as a targeting ligand thanks to its high specificity toward ER+. To add the EE2 to VLP, first a chemical substitution was realized at the ethynyl position 17 of EE2, which retained its bioactivity, and thus, an amine functionality at the ethynyl position of EE2 was introduced by Sonogashira coupling with 4-bromoaniline, producing an estradiol derivative, ESTAm. For the covalent conjugation of ESTAm on the VLP surface, a heterofunctional PEG with succinimidyl ester and the maleimide group (NHS-PEG4-Mal) at the distal ends was used, while the NHS group enabled a selective conjugation of ESTAm via amide bonding (forming PEG-EST). The maleimide site was covalently attached to the exterior of VLPs. After 12 h of culture with nanoparticles, the ER+ cells showed the preferential intracellular and endosome localization of P22CYP-PpIX-PEG(EST) particles, inducing the specific delivery of VLP thanks the estradiol molecule. The use of PEG also enabled the control over the nonspecific cellular uptake and most importantly reduces immunogenic response of nanoparticles by providing stealth properties. An immunological analysis confirmed that the presence of PEG moieties on the VLP surface significantly brings down the activation of the NFkB and AP-1 reporter genes in macrophages, two transcription factors that play central roles in inflammation and immunity. The toxicity analysis showed that the untargeted nanoparticles presented nearly the same cell viability as the tamoxifen free treated cells (~74%), probably owing to the poor cellular uptake. Additionally, the targeted P22CYP-PEG(EST) and P22CYP-PpIX-PEG(EST) increased the tamoxifen sensitivity by ~twofold (~38% of cellular viability) by EPT. Further, the combination of EPT and PDT using P22CYP-PpIX-PEG(EST) caused ~24% of the cells to be viable, decreasing to ~16% when doubling the particle’s concentration, which is similar to the positive control (DMSO), showing a highly efficient tumor treatment that demands a lower concentration of the administered drug [69]. These results illustrate the ample variety of chemical linkages that can be used for particle decoration. However, another amply used form of particle decoration is the genetic insertion of the non-native structures into the VLP.

Generally, the direct genetic insertion of structures onto the VLP outer surface is carried out to insert peptide sequences capable of conferring specificity to a determined target. One example is the insertion of the RGD (arginine-glycine-aspartic acid) tripeptide to P22’s coat protein (CP), which interacts specifically with the α_V_β_3_ integrin, a cell receptor that is overexpressed in certain cancer cells [51]. The peptide was inserted at the threonine 183 (T183) residue of the CP A-domain, the outermost region of the P22 capsid. Such a modification would be ideal for the targeted delivery of therapeutics against cancer, but it is important that it not compromise the VLP structure. Although the RGD insertion was permissive to the particle assembly of the PC particle, it interfered with the structural transition capabilities of the VLP, increasing the necessary energy to induce the expansion to the EX form. The expansion was possible by heating the particles at 67.5 °C and 70 °C to induce the transition of ~60% of particles compared with those heated at 65 °C. Moreover, the peptide completely impeded the obtention of the WB because particles decomposed upon heating at 72 °C [51]. Therefore, it is important to consider the inserted structures properties because there is a limit to the size and types of structures that can be incorporated through this method without compromising the particle structure. Therefore, the development of alternative approaches for the stable incorporation of large structures is of particular interest in VLP design. Examples of such strategies will be discussed below and include the following: the genetic modification of the CP subunits to include peptide sequences for the covalent binding with the desired structure [12,17,100] and the usage of the P22 VLP electrostatic properties for the noncovalent binding of structures to the particle surface [60,61,62,92,102].

The genetic modification of the CP primary sequence through the insertion of non-native peptide sequences can be used as a base for the implementation of other strategies [12,17,100]. One such example involved the exploitation of simple peptide sequences that could easily be inserted at the C-terminus of the CP subunits. It served as a base for the binding of larger structures through the formation of covalent peptide binding mediated by sortase, between the VLP surface and the protein of interest [17]. Sortase is an enzyme capable of catalyzing the binding of a protein that possesses a LPETG residue sequence in its C-terminus to a protein that presents a polyglycine sequence in its N-terminus. The reaction occurs between the threonine (T) residue of the sequence and the glycine, permitting the sortase-mediated covalent binding of structures to the VLP surface by genetically inserting the LPETG peptide into the C-terminus of the CP subunits. This sequence did not affect particle assembly or its expansion capabilities. This approach is favorable to the incorporation of large proteins into the VLP exterior because the addition of polyglycine markers to the target protein is not as invasive as the insertion of the peptide. Through this technique, the annexation of large molecules, such as the globular head domain of the influenza hemagglutinin (HAhead) protein to the P22 VLP, becomes viable. Using recombinant fusion, polyglycine sequences were inserted into the N-terminus of both the HAhead and green fluorescent protein (GFP) for sortase-mediated conjugation to the particle surface [17]. In order to promote binding, PC and EX VLP-LPETG particles were incubated with different concentrations of the glycine-altered proteins. Initially, small-scale procedures with glycine-modified GFP were used to probe the binding of proteins to the VLP exterior, where density analysis reported a 15% binding efficiency, indicating a GFP and VLP-LPETG reaction with a proportion lower than 1:1. This low yield is not surprising, in that the proximity of the C-terminus of the capsid subunits to the fivefold and sixfold symmetry centers, in addition to the binding of the initial GFP molecules to this binding site, can result in steric clashes between the annexation of the subsequent GFP units. Approximately 140 GFP copies were bound to the capsid, the equivalent of almost 2 GFP for each symmetry center on the P22 capsid. When a larger-scale reaction was carried out, there was an increase in the number of GFP molecules bound per capsid to 176. Interestingly, in these larger-scale tests, the formation of a solid disk structure was observed when the samples were submitted to cesium chloride ultracentrifugation, which was composed of multiple particles. It was theorized that the disk formation was a result of the interactions between the GFP units present at the particle’s surface, which generated oligomers, which in turn led to the formation of the disk structure. After testing using GFP, the sortase-mediated conjugation of the HAhead was investigated. When analyzing SDS-PAGE band density, it was estimated that close to 183 HAhead molecules bonded to the capsid, which was consistent with the presence of two protein subunits in each axis of symmetry. Different from GFP, no disk-like structure was observed [17]. These results present a strategy to covalently attach relatively large structures to the P22 VLP exterior.

Another approach for the decoration of the P22 VLP with large molecules is the use of the bacterial superglue derived from the *Streptococcus pyogenes* fibronectin ligand FbaB, the post-translation binding system SpyTag/SpyCatcher (ST/SC) [12,100]. This system consists of the use of split proteins capable of forming an isopeptide bound in a selective and fast way, occurring on the basis of the variation in pH, buffer, and temperature when the proteins are mixed together, without the additional use of chemical components or enzymes. It works by genetically fusing the ST to the C-terminus of CP, exposing it to the external environment and allowing it to be recognized by and interact with the SC genetically fused to the desired structure. One study used this system to decorate P22 VLP with ligands and test the targeted delivery of an anticancer drug [12]. The ST/SC system was used to decorate the external surface of P22 WB VLP with either an epidermal growth factor receptor (EGFR) affibody (EGFRaf) or a human epidermal growth factor receptor2 (HER2) affibody (HER2af), and both ligands that can be used to target breast cancer cells by binding with its specific receptor. Simultaneously, the doxorubicin (Dox) prodrug 6-maleimidocaproyl hydrazine, or Aldoxorubicin (AlDox), was covalently linked to either the interior or exterior of the WB VLP through a thiol-maleimide coupling reaction into the inserted cysteine residues (S133C or K118C, respectively). AlDox possesses a hidrazin crosslink between Dox and its maleimide moiety that can be subsequently cleaved under acidic conditions, resulting in the release of cargo to the surrounding environment, allowing it to penetrate the tumor cell nuclei and disrupt the genomic DNA and/or its repair system.

Through F5M labeling, followed by fluorescent microscopy, both the binding and the internalization of the VLP particles by the target cells was confirmed. The cytotoxic effect of this construct against breast cancer cells was evaluated through MTT assay, comparing it with free Dox. It showed an efficacy similar to or even higher than free Dox, indicating that the directed delivery of Dox resulted in an increase in the cytotoxic activity. The possible occurrence of side effects and of nonspecific binding was also analyzed by treating MCF-7 and MCF-10A cells that do not overexpress the EGFR or the HER2 receptors, respectively, with free and entrapped Dox. While the cells treated with free Dox showed similar results to other tests, those incubated with loaded VLPs expressed only basal levels of mortality. According to these results, targeted-delivery-mediated P22 VLP could be an efficient form of cancer treatment with minimal side effects [12]. Both previously mentioned methods are efficient in incorporating large structures into the particle surface but require the direct modification of the capsid structure. There are other ways to add structures to the P22 VLP surface that do not require the modification of its structure and that exploit its native properties.

An interesting feature of the P22 VLP that sets it apart from other VLPs is its capability to exploit the inherent electrostatic properties of the capsid in the noncovalent incorporation of exogenous structures [60,61,62,102]. The decoration of the particle with large proteins has been previously described through the use of the phage L decoration protein (Dec), a trimeric protein that has shown an elevated affinity to the P22 EX capsid surface selectively binding to it. It was shown to bind specifically to two sites of the capsid with varying intensity, the quasi-threefold symmetry axis and the true threefold axis, which have higher affinity for the first one [61,62]. There was a reported total of 80 potential binding sites per EX particle, 60 at the quasi-threefold sites and 20 at the true threefold sites, resulting in a total of 240 Dec monomers [61]. The interaction occurs between the N-terminus of the Dec subunits and the C-terminus of the capsid proteins, making it an advantageous site for the annexation of exogenous structures. This is achieved by genetically manipulating the Dec structure, as reported in the first study that used the inserted 6x-His-Tag or a cysteine residue to bind cargo to Dec monomers [61]. The insertion of the marker into either terminus of the Dec monomer did not impede trimerization or binding to the P22 VLP surface. Through the gold labeling of the Dec protein C-terminus that faces the exterior environment, it was observed that the protein extended outward a distance of ~95 Å from the particle surface [61]. This position is considerably accessible and is less likely to suffer from steric hindrance, making it ideal for molecular presentation. The use of Dec protein for such an end is interesting given that it does not require or result in a significant change in the P22 morphology, allowing the incorporation of large cargo that could not be directly genetically inserted. One such study tested the incorporation of a 21-amino-acid-long peptide (Self), derived from CD47, or the 17 kDa soluble region of murine CD40L, into VLP particles containing SP-mCherry as cargo [62]. The CD40L (CD154) is a transmembrane cytokine with a TNF-like trimeric structure capable of activating adaptive immunity and whose presentation using the trimeric Dec structure could significantly increase its triggering capabilities. Its 148-amino-acid-long soluble region was successfully incorporated into the Dec protein C-terminus, where the construct subsequently bound to the particle surface. The VLPs decorated with the Dec-CD40L constructs were capable of binding to primary murine B lymphocytes cells in an increased manner when compared with particles lacking the CD40L, as assessed by fluorescence-activated cell sorting. The increase in internalization indicates that the CD40L retained its activity, similarly to what was observed from the Self peptide. The Self peptide is capable of binding to the SIRP-R receptor-inhibiting phagocytosis by macrophages, and thus, the decorated particles would be capable of evading internalization. The peptide was also fused to the C-terminus of the Dec protein monomers and successfully decorated the particle surface. When particles were incubated with splenocytes, it was observed that the decorated particles had decreased cellular uptake compared with the particles lacking the peptide, which were close to control levels. This indicates that the decorated Self particles had minimal cell interactions, which supports the claim that Dec presentation does not result in a loss of function [62]. In addition to the presentation of target proteins, Dec protein decoration can be explored for other applications in VLP design, such as reinforcing the particle structure. As cement proteins such as Dec naturally act as structural reinforcements for the capsid, it is no surprise that Dec decoration can increase P22 VLP stability. External decoration with Dec reportedly increased the stability of different P22 VLP morphologies, even the WB, in which its pentonless nature could make it more fragile [60]. Another paper has also described the use of Dec protein for interparticle binding applied to the design of supramolecular structures [102].

The natural surface charge of the P22 capsid can be exploited for the annexation of cargo without the Dec protein: another study used the external charge of VLP to synthesize the controlled size of gold nanoparticles (AuNPs) [92]. By simple mixing the VLPs with HAuCl4 (gold precursor) in deionized water, the gold ions electrostatically interact with the negatively charged histidine residues, directing the nanoparticles’ growth in a spherical face-centered cubic structure (fcc). The AuNPs’ sizes can be controlled by the gold precursor concentration added to the sample, reaching from 2.8 nm (35 mM of precursor) to 8.7 nm (175 mM of precursor). Thus, these results show that the P22 surface can be explored for the incorporation of numerous types of structures, some of which are listed in Table 2, further expanding the number of possible applications.

## 6. Use as a Nanocarrier

One of the most studied applications of VLPs is its use in the targeted delivery of pharmacological cargo for the treatment and bioimaging of diseases such as cancer. The delivery of therapeutic agents mediated by VLPs is one the most widespread applications of this technology because it significantly heightens the agents’ efficiency [12]. By using VLPs for the targeted delivery, it could be possible to reduce the side effects of aggressive treatments, such as chemotherapy, that significantly lower the patient’s quality of life. It also has the potential to facilitate drug access and the treatment of particularly delicate regions of the body such as the central nervous system (CNS), easing the treatment of diseases such as glioblastoma, a malignant cancer arising from astrocytes. This cancer is characterized as a grade IV glioma and is one of the most aggressive and invasive cancers affecting the CNS. The life expectancy of a person diagnosed with this type of cancer is on average 12–15 months. Its main treatment consists of chemotherapy, with extreme cases requiring the surgical removal of the tumor, and even so, survival rates are minimal. In trying to come up with a less aggressive approach through a more specific action to target drugs in the treatment of glioblastoma, our research group considered the use of VLP P22 as a nanocarrier of drugs for the central nervous system.

We tested the potential application of the P22 VLP as a drug nanocarrier for CNS diseases, with an initial focus on glioblastoma. The methodology [16,105,106,107,108] and results are shown in the Supplementary Materials. Particles containing the scaffolding protein (SP) mCherry were obtained from heterologous protein expression in *E. coli*. In order to test selective particle incorporation, experiments were conducted using 0.5 mg of VLP that was incubated for 2 h with glioblastoma cells, normal astrocytes, and neurons. Fluorescence microscopy was used to confirm VLP internalization (Supplementary Figure S1). The cell cytoskeleton was labeled with Alexa 488 fluorophore-tagged antibodies and identified by the characteristically green fluorescence, while VLPs containing the fluorescent protein mCherry emitted red fluorescence. Cell nuclei were labeled with DAPI (4,6’-diamidino-2-phenylindole, dihydrochloride; 4’,6-diamidine-2-phenyl indole; 2-(4-Amidinophenyl)-6-indolecarbamidine dihydrochloride), emitting blue fluorescence. Our results showed the incorporation of P22 VLPs in primary glioblastoma cells (Supplementary Figures S1 and S2). In contrast, such incorporation was not observed in neuron cells (Supplementary Figure S3). This is a very important finding for further studies. Neurons are cells that have a low capacity for replication, and consequently, damage to this cell type can become irreversible and represents a great danger to the patient. Therefore, the lack of particle incorporation by the neurons can make this approach an excellent candidate for drug delivery to the central nervous system to reduce the risks of treatment with chemotherapy. The binding of VLPs has also been tested in astrocyte cells, which are the healthy cell types from which glioblastomas originate. Confocal fluorescence microscopy images confirmed the internalization of particles in this cell type, as had been seen for glioblastoma and absent in neurons (Supplementary Figure S4). The results with astrocytes can guide two main lines: the first would be to increase the specificity for cancer target cells, avoiding affecting healthy cells. The second would be related to the use of these VLPs in initial treatments, whose target is astrocyte cells in the primary stages of cancer development. As an alternative to modulating the incorporation of P22 VLPs into target cells, the use of cell-penetrating peptides (CPPs) conjugated to the viral particle was considered. From this, in silico predictions were performed to evaluate possible candidates for CPPs in the bacteriophage P22 protein sequences (Supplementary Figure S5). Our predictions relied on the following software: CPPred, CellPPD, SkipCPP, and eMCPP [109,110,111,112]. Using the sequence of all proteins with a known function for bacteriophage P22, obtained from the NCBI database, 10 amino acid peptide sequences were searched to find possible candidates for CPPs. After determining the possible adequate peptides, their sequences were altered in order to include a cysteine residue at either its C-terminus or its N-terminus, followed by new predictions. The inserted cysteine would allow it to be used for crosslinking to the VLP surface. Through this, we were able to determine the possible peptides sequences that could be used as CPPs, as shown in Supplementary Figure S5.

CPPs have already been proven to significantly expand VLP potential. The incorporation of CPPs confers the capacity of surpassing biological barriers that tend to hinder drug distribution and action, one such example being the brain–blood barrier (BBB). Therefore, using it to decorate the VLP is ideal for the delivery of fragile cargo that has a low accessibility capacity, such as peptides. One example of P22 VLP’s capacity for the delivery of such cargo was when the therapeutic peptide MVIIA was encapsulated into a CPP-decorated particle [56]. This peptide has analgesic properties and is composed of 25 residues expressed in the venom of the marine snail species *Conus magus*. It has been explored in the treatment of neuropathic pains thanks to its being a nonopioid substance that can act specifically on the type-N calcium channels, avoiding the opioid receptors, therefore lacking the drawbacks of such drugs. Its application is limited owing its dependence on the integrity of its rigid structure, rich in disulfide bridges, in a way that any modification can result in loss of component efficiency. Moreover, its systemic application is hindered by a low permeability that makes it unable to bypass certain biological obstacles. Thus, its encapsulation inside the CPP-decorated P22 VLP through its genetic fusion to the truncated SP (238–303) can highly improve its efficacy [56]. Particle assembly and cargo internalization were analyses through transmission electron microscopy (TEM) and multiangle light scattering (MALS). The change in weight observed by MALS indicated the presence of 543 copies of the chimeric compound in the particle interior, which is in a much higher number than the number of copies that normally participate in assembly or the estimated canonical binding sites. This greater number of fusion copies is in accordance with the previously discussed observation that the truncated SP is capable of nonspecific binding to the particle’s interior [13].

In order to be able to bypass the BBB, the P22 VLP has been decorated with the penetrating peptide HIV-Tat. Through the substitution of a methionine residue with a cysteine (M339C), a functional group was introduced, one that would later be used for the incorporation of the CPP through chemical modification. The HIV-Tat was labeled with a fluorescein marker (FAM) and incorporated into the VLP surface through a chemical reaction to the cysteine. The change in band migration and the density of SDS-PAGE, when compared with the native particle, confirmed the modification, where it showed a molecular weight indicative of the capsid–peptide fusion, and its density suggested that close to 40% of the CP subunits were conjugated to the peptide.

The VLP-HIV-Tat capacity to surpass the BBB was initially evaluated by using an in vitro model of the barrier composed of a monolayer of rat brain microvascular endothelial cells (RBMVECs), a model commonly used for drug transposition through the barrier. Using this system, an efficient cell uptake was confirmed after 20 min by the observation of green fluorescence, emitted by the FAM marker, in the cell’s interior. To further confirm and characterize the VLP construct penetration capacity, a dynamic BBB in vitro model composed of human microvascular endothelial cells and human astrocytes was used. The particles were applied to the luminal chamber of this model, and the translocation was evaluated by measuring the transendothelial electrical resistance across the barrier cells to the extracellular space of the endothelial cells. After 15 min, ions referring to the VLP particles initially present on the lumen chamber were detected in the extracellular space, indicating that the particle had started its passage.

In order to evaluate the biocompatibility of the construct, a colorimetric MTT assay was used through the 24 h incubation of the RBMVECs, with increasing concentrations of VLP loaded with MVIIA peptides. Treatment with the particle did not cause cytotoxicity to the cells, even when treated with double the concentration of particles used for all experiments, as observed by the similarity in the viability of treated cells when compared with the untreated ones. To further characterize its biocompatibility, the particles were intravenously injected into nude mice that had malignant subcutaneous tumors. The particles were injected into the tail of the animal, and its arrival at the tumor was confirmed by the ex vivo epifluorescence analysis of the organ at different intervals. The high incidence of fluorescence in the excised tumor confirmed the successful traffic of the VLP through the mice, and although it did not confirm its internalization by the central nervous system, it did show that the construct was not degraded in the blood [56].

Another example of therapeutic cargo that has already been delivered using P22 VLP is bortezomib (BTZ), a dipeptide with anticancer properties that was loaded in the WB P22 for the targeted delivery against hepatocellular carcinoma cells (HCCs) [9]. BTZ has a boronic acid moiety that interacts with threonine residues in protease-active sites, and this moiety is capable of inhibiting the proteasome inside cancer cells, leading to cell death and inhibiting tumor growth. BTZ was incorporated into the interior of the WB P22 modified with N-(3,4-dihydroxy- phenethyl)-3-maleimido-propanamide (Catechol-Mal) ligands. These ligands were covalently conjugated to an inserted cysteine residue at position 118 through a thiol-maleimide reaction with a Michael-type addition. Catechol ligand was chosen because the boronic acid moiety of BTZ can form a stable bond to it under neutral or alkaline pH through a boronic acid–diol complex that can be easily dissociated when submitted to acidic conditions, resulting in the release of BTZ, allowing it to act on the cancerous cells. To evaluate the potential for the targeted delivery of BTZ mediated by the WB, the VLP was decorated through chemical crosslinking with the peptide SP94, which confers specificity to HCCs. The cytotoxic potential of the construct was evaluated through a MTT assay where the cells were treated with the BTZ-loaded and SP94-decorated WB particle; with the loaded particle but lacking the targeting peptide; with the unloaded and decorated particle; and with free BTZ as the positive control. After 18 h, it was observed that the particle modified with the peptide ligand and carrying BTZ had almost the same effectiveness as the free drug, each achieving close to 80% and 90% of cell death, respectively. Empty VLP particles did not show an expressive percentage of cell death, while the loaded particles that lacked the peptide ligand had a significant reduction in cytotoxicity, with only ~40% effectiveness. Through fluorescence microscopy, not only was it confirmed that the SP94 ligand binds to the cell, permitting the VLP to also adhere to it, but it was also suggested that it also allows particle endocytosis because the presence of particles surrounding the cell nucleus was observed. These results show that the WB modified with SP94 can promote an efficient delivery of BTZ with a concentrated and local release both on the surface and in the interior of tumor cells, with a pH-dependent release strategy [9]. This pH-dependent release strategy is interesting because certain medically relevant sites have characteristic properties that can be exploited, such as the acidic environment of tumors and the interior of endosomes [9,12,85]. A similar strategy was used for the targeted delivery and release of a combination of therapeutic peptides [85]. In addition to being used as therapeutic agents, VLPs can also be used to safely carry molecules with toxic potential.

The use of contrast agents (CAs) in magnetic resonance imaging (MRI) is limited by the toxicity of these molecules, such as gadolinium(III), which can injure nephrogenic systemic fibrosis in patients with renal failure, and is limited by the decreasing of their ionic relaxivity in highly magnetic fields [93], which is the capacity to increase the proton relaxation rate, strengthening the MRI signal. The encapsulation of fluorescent molecules or CAs can enhance the function of these molecules in MRI thanks to their high concentration per volume, which strengthen the imaging signal, increasing sensitivity and resolution of the analysis. In this way, it is possible to turn the P22 VLP into a highly efficient bioimaging complex. Higher fields offer a greater signal-to-noise ratio, a better spatial resolution, and reduced acquisition times, which have clear benefits for many applications, including functional brain imaging and spectroscopy. Gadolinium-based small molecules and manganese(III) protoporphyrin IX (MnPP) complex are examples of CAs highly used in medical imaging protocols. Aiming to avoid the contact of Gd with the environment and create a safe and efficient CA, Lucon et al. packaged gadolinium diethylenetriaminepentaceate-Gd(DTPA) into VLPs. They used a 2-aminoethyl methacrylate polymer (xAEMA) as a crosslinker and tested the encapsulation with (P22S39C-xAEMA-Gd) and without xAEMA (P22S39C-int-Gd). The xAEMA promoted an increase of 28 times the number of Gd/capsid, showing that the electrostatic interactions between the cargo and the P22 capsid are nonrelevant and that the majority of the Gd(DTPA)-NCS molecules are bonded to the polymer, which enables control of the loading factor by controlling the quantity of polymer inside the particle. Moreover, for Mn(III), the results showed evidence of noncovalent interactions between MnPP and P22, but the loadings with a covalent ligand were much higher, and they could control the number of molecules per VLP by repeating the encapsulation process. The first reaction resulted in 780 molecules/capsid, and this number increased to 1200 and 3646 MnPP molecules/capsid during the second and third reactions, respectively. The ionic relaxivities of Gd-DTPA encapsulated with and without the polymer were higher than those free of an environment. Thanks the high load, P22S39C-xAEMA-Gd presented a particle relaxivity one order of magnitude higher than that of the other macromolecular assemblies, indicating that the encapsulation improves the CAs’ bioimaging applications [78]. Additionally, both P22-Gd(DTPA) and P22-MnPP can be used as CAs for a positive contrast (T1) or a negative contrast (T2). This can be controlled by the payload of VLP or the magnetic field strength [93,94]. Usselman et al. showed that the highest loaded sample (10,300 Gd(III)/VLP) acts as T1 at low fields and as T2 at high fields [94].

## 7. Cargo Release Strategies

**Figure 7:**
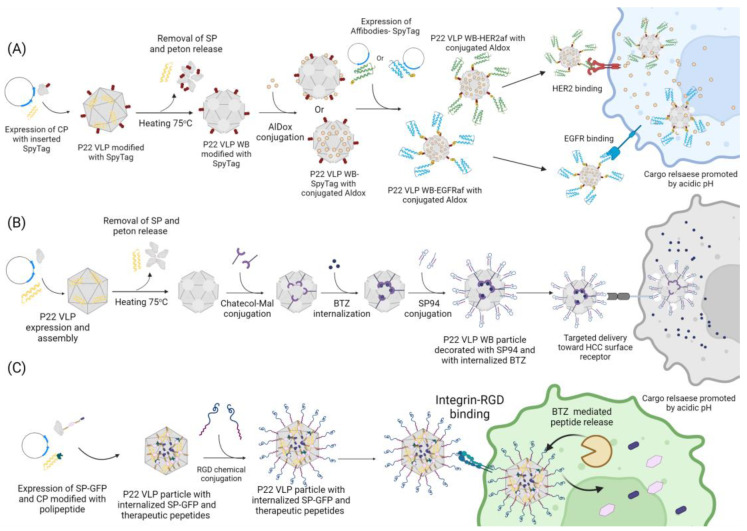
Targeted delivery of cargo. The scheme illustrates some examples of the reported methods for the targeted cargo delivery and release inside the cell. (**A**) The expression of CP subunits containing the inserted SpyTag allows for the decoration of the P22 VLP WB particles with large molecules, such as affibodies for EGFR (EGFRaf) and HER2 (HER2af) modified with the SpyCatcher sequence. These modifications allow for the targeted delivery of Aldoxorubicin (AlDox), which can be bound to the WB particle interior/exterior surface through thiol-maleimide coupling with inserted cysteine residues (S133C or K118C, respectively). The ligands will interact with the particle’s specific receptor, promoting the particle’s endocytosis, and once inside the cell, the acidic pH will promote the cleaving of AlDox’s hydrazine crosslink between Dox and its maleimide moiety, resulting in the drug’s release [12]. (**B**) Another pH-dependent release strategy involves the use of a catechol-mal ligand to incorporate BTZ into P22 WB particles and release the dipeptide when submitted to low-pH conditions. After BTZ internalization, the loaded P22 WB particles were chemically modified with the peptide SP94 to HCCs, as the ligand binds to surface receptors of the tumoral cells. After internalization, the acid–diol complex that formed between BTZ’s boronic acid and catechol-mal’s maleimide moieties is undone, resulting in the release of the drug inside the cell [9]. (**C**) Other methods for the environmentally triggered release of cargo can exploit enzymes to propitiate drug exit, one example using the lysosomal cysteine CTB to promote the release of peptide cargo from within the P22 VLP after endocytosis. The NuBCP-9 and the KLAK therapeutic peptides were encapsulated in vivo into the P22 VLP by being inserted into the N-terminus of CP subunits and coexpressed with SP-EGFP fusion. The peptides were incorporated in an intermittent way with CP, and both therapeutic peptides were intercalated with linker sequences that can be cleaved by CTB. The loaded P22 VLP was chemically modified with cyclic RGD peptide, conferring specificity to αVβ3 and αVβ5 integrins. Once inside the cell, CTB cleaved the linker sequences and released the peptides into the cell [85].

Although many studies have tested and shown the methods of cargo internalization, particle modification, and directed delivery using P22 VLP, there are still few reported strategies for cargo release once the targeted location has been reached. However, the examples published describe different and versatile ways for cargo release. Among the most used strategies for the release of cargo is using the environmental pH of the particles’ location as a trigger. As previously described, the use of an acidic pH for the release of cargo is in recurrent use thanks to the low pH in the cells’ interiors and thanks to the tumoral environment [9,12]. One approach used the SpyTag/SpyCatcher (ST/SC) system to modify P22 VLP wiffleball (WB) particles with either an epidermal growth factor receptor affibody (EGFRaf) or a human epidermal growth factor receptor2 affibody (HER2af) to target breast cancer cells and deliver the prodrug Aldoxorubicin (AlDox) (Figure 7A) [12]. The molecule was covalently linked to either the interior or the exterior of the WB VLP through thiol-maleimide coupling and was released by the pH-induced cleaving of AlDox’s hydrazine crosslink between Dox and its maleimide moiety [12]. The other used WB particles decorated with the peptide ligand SP94 for the delivery of the dipeptide bortezomib (BTZ) to hepatocellular carcinoma (HCC) cells (Figure 7B) [9]. BTZ was incorporated into the interior of the WB P22 by binding it with catechol-maleimide (Catechol-Mal) ligands through the formation of a stable bond with the boronic acid moiety of BTZ, which can be easily dissociated when submitted to acidic conditions, such as the cells’ interiors. Both methods released the cargo through the P22 VLP wiffleball (WB) particles’ pore (or the exterior in the AlDox case) after the pH-induced cleavage of the ligand molecule, and they resulted in the effective elimination of the targeted cell, confirming the drugs’ release. In addition to exploiting pH levels, other elements of the cell environment can be used to promote cargo release, such as enzymes, as described below.

Cathepsin B (CTB) is a lysosomal cysteine that is stably regulated under normal physiological conditions but upregulated in cancer cells, making it interesting for exploitation for the enzyme-mediated release of drugs. One paper reported its use as a trigger for the release of therapeutic peptide cargo after targeted delivery using P22 VLP (Figure 7C) [85]. The NuBCP-9 (FSRSLHSLL) and the KLAK (KLAKLAKLKALAKL) therapeutic peptides were loaded into the P22 VLP by being inserted into the N-terminus of CP subunits. The NuBCP-9 peptide is capable of binding to BCL-2 and can act as a switch capable of inducing conformational change in BCL-2, converting it into a killer and inducing apoptosis. The KLAK peptide is composed of a 14-residue polycationic chain capable of disturbing the mitochondrial chain triggering an apoptotic response in eukaryotic cells. Together, the peptides can have a synergic effect to eliminate the cells acting mainly on the mitochondria. Both peptides were fused to CP to ensure a well-defined quantity of loaded peptides. The peptides were incorporated in an intermittent way with CP, and both therapeutic peptides were intercalated with linker sequences (GFLG) that can be cleaved by CTB. Furthermore, to track the peptides’ traversal in the target cells’ interiors toward the mitochondria, the FLAG-tag sequence (DYKDDDDK) was fused to the NuBCP-9 N-terminus, and an anti-FLAG-tag antibody was used to visualize the intracellular traffic through immunofluorescence. Aiming to attenuate the possible adverse electrostatic effects that the repulsion between the highly positive peptide cargo could have on particle assembly, SP that was fused to the negatively charged, enhanced green fluorescent protein (EGFP) was used. Chemical modification was also used for the annexation of cyclic RGD peptide (cyclo [Arg-Gly-Asp-d-Phe-Lys]-(12-Ado)-(PEG12)-(6-Mal)) to the P22 VLP exterior by binding to mutant cysteine residue (M338C). This modification was used to confer specificity to αVβ3 and αVβ5 integrins, which are overexpressed in the breast cancer cells (MD-MBA-231) used in the study. Confocal laser scanning microscopy (CLSM) fluorescence imaging confirmed the internalization of P22 VLP particles and their presence in the interior of lysosomes, making them susceptible to the action of proteases. Treatment with chloroquine was used to improve the traffic efficiency of the endocytosed cargo, and immunofluorescence imaging confirmed that the peptides arrived at the mitochondria. The cytotoxic effect of the delivered peptides was confirmed through cell counting kit-8 (CCK8) assay and flow cytometry with significant inhibition on cell growth (*p* < 0.05), thus proving the P22 VLP potential for peptide delivery and cargo release through an enzymatic trigger [85]. The previously described strategies for cargo release used environmental conditions as triggers, but recently, methods for the controlled liberation of cargo have been described.

One of the most interesting methods for the release of cargo proposed the triggering of particle disassembly for the release of cargo in physiological pH under a defined and controllable stimulus, which did not rely on environmental conditions [76,77]. The strategy consisted of the chemical modification of the P22 VLP structure by conjugating Norbornene (5-Norbenen-2-Carboxylic acid) to exposed lysine residues on the particle’s exterior. Norbornene is a cyclic olefin that can be used as a substrate for a ring opening polymerization reaction (ROMP) when in the presence of a catalyst. Once the reaction occurs, it triggers the polymerization of Norbornene, which consequently disturbs the VLP structure, promoting particle disassembly. This method of particle disassembly is particularly interesting for the development of P22 VLP as a nanoparticle thanks to its allowing for the timely release of cargo through a controlled stimulus and under physiological conditions. Thus, whether the P22 VLP could have its exterior modified with Norbornene and its particle disassembled to release in internalized cargo was investigated, where SP-GFP fusion was used as the cargo [76]. The decoration of the P22 VLP’s external surface was performed through chemical conjugation by activating the Norborne-COOH with EDC and sulfo-NHS, which induced the formation of an amine bonding with the particle-exposed lysine residues. Mass spectrometry analysis was used to monitor the conjugation reaction and confirmed the successful surface modification. The ROMP reaction as triggered by using a second-generation Grubbs catalyst (Grubbs II catalyst), and it confirmed the disassembly of the VLP particle through transmission electron microscopy. This initial study confirmed the potential of using Norbornene decoration and ROMP reaction for the triggered disassembly of P22 VLP, without being restricted to environmental conditions. However, the effective release of cargo would be confirmed only in a subsequent study, where the highly water-soluble AquaMet catalyst was also tested as a trigger [77]. In this new research, the release of the SP-GFP cargo was evaluated through fluorescence assay, which evaluated the changes in fluorescence in the aliquots of particles treated with AquaMet catalyst. Particle disassembly and cargo release were confirmed by the analysis of absorbance at 280 nm (relative to the aromatic residues present in CP) and 495 nm (relative to GFP). The observation of a significant absorbance peak in the aliquots of the treated particles indicated both particle disassembly and cargo release [77]. These results present a strong indication of the potential to exploit the ROMP reaction as a strategy for the controlled release of cargo, although it has yet to be tested in the delivery of therapeutic cargo.

## 8. Vaccine Platform

One rising use for VLPs is as vaccine models, which have presented some advantages over the other vaccines [113,114]). The lack of genomic material and proteins responsible for virus replication make the VLP vaccines safer than inactivated or attenuated virus vaccines. The subunit vaccines have similar properties, but the protein’s production in the absence of other virus components causes a proportion of the subunits to have aberrant conformation when compared with that of the native protein. This consequently hinders their immunogenicity and can compromise memory induction. Studies have shown that they do not infect the antigen-presenting cells, so the memory killer T cells are not induced [115,116]. The VLP is more efficient than the subunit vaccines because it mimics the overall structure of the virus and the proteins at the native conformation and can stimulate both cellular and humoral immune responses. In particular, spherical or icosahedral VLPs are more efficient in displaying antigens because of the high density and homogeneous distribution of the presented antigens. Previous studies on a Zika virus vaccine compared the VLP and the inactivated virus. Both produced high levels of IgG antibodies, but the Zika VLP stimulated higher neutralizing antibody titers, thus illustrating the potential that VLPs can have as vaccine models [114]. The P22 VLP has also been explored for the development of vaccine platforms. The first study of this kind explored the particle immunomodulatory agents for lung priming [117,118]. The capacity of P22 VLP to incorporate multiple cargoes in vivo through the genetic manipulation of the SP protein has already been used to internalize and present the nucleoprotein of the influenza A virus [83], as well as a poliantigen composed of respiratory syncytial virus matrix proteins [79]. In addition to interior loading, the exterior surface of the P22 VLP was also explored for antigen presentation. The complexity of some viruses may make the production harder and more expensive. On the other hand, an external antigen can be attached to the surface of the P22 VLP, turning it into a vaccine platform that is easier to produce for diverse viruses. One paper reported on the presentation of hemagglutinin head domain (HAhead) on the P22 VLP surface mediated by the SpyTag/SpyCatcher (ST/SC) system [100]. Another study tested the ability of fusing peptide molecules to the P22 VLP CP in order to present ovalbumin epitopes T and B, in order to develop an antitumoral vaccine [101].

## 9. Nanoreactor

The hollow interior of the VLP creates a very restrained space that can be explored for the study and development of contained reactions, especially enzymatic ones. The sturdy protection that the VLP structure confers to cargo is particularly interesting for the delivery of enzymes thanks to the fact that its functionality is dependent on the proper folding of its structure. Furthermore, the confined space provided by the capsid can be explored to study the impact of molecular crowding in enzyme activity. Thus, the P22 VLP has been extensively explored for enzyme transport as a nanocarrier, releasing the enzymes in the environment, or as a nanoreactor, when the reaction occurs inside the VLP. The high number of confined enzymes can increase the velocity of reactions. This construct can be used in medicine, such as for diseases that originated from the lack of enzymatic activity, cancer treatment, and antimicrobial applications [69,70,71,72], or in the fuel industry by hydrogen production [68]. Most studies on this subject utilize in vivo assembly by heterologous expression with the enzyme genetically fused to the scaffolding protein (SP), which have a well-established and easy-to-reproduce protocol. Different strategies to obtain the maximal enzyme activity have been reported [66,68,70]. However, a highly crowded environment may sometimes interfere with enzyme activity [69,70,71]. To circumvent this issue, several groups have utilized coexpression with different plasmids, with a later expression of coat protein (CP) to control the individual expression, increase the number of encapsulated enzymes, and give time for the maturation of the enzymes.

The previously mentioned cytochrome P450 (CYP), an enzyme from *Bacillus megaterium* that was encapsulated through genetic fusion to the scaffold protein (SP), can act in two ways: first by the production of ROS inside the tumor cells and second by the activation of the tamoxifen, a classic prodrug used in some types of cancer [69,71]. Sánchez-Sánchez et al. [70] managed the highest CYP loading (156 molecules per particle), close to the theoretical maximum (180 molecules per particle), but an inactivation of 93% of the enzymes was observed, which could have been a result of the fast interaction of CYP with CP. The solution was to use different plasmids for CYP-SP and CP expression that allowed for the control of inductors’ concentration and induction times, which increased the percentage of the active enzymes to 35%, with little reduction in the cargo density (109 molecules per particle). The encapsulated CYP showed half the individual catalytic activity of that of the free enzymes, but the treated cells resulted in 10-times-higher CYP activity than that of the untreated cells [70]. When using the tamoxifen technique, a lower individual catalytic activity for the encapsulated enzyme was observed compared with the free enzyme, but the viability of cells treated with P22-CYP decreased, with half the drug concentration compared with the untreated cells [71]. The reduction in the administered drug quantity led to reduced side effects associated with hepatic dysfunctions and diseases, which shows the high potential of the VLP for such an application.

Qazi et al. [57] encapsulated CRISPR-Cas9 molecules, a large molecule used as a biological tool with the potential to create sequence-specific genome modifications. As before, SP-CRISPR and sgRNA + CP were expressed with different promoters. This system also allows for the temporal decoupling of cargo and capsid expression. A delayed expression of CP facilitates the correct assembly of cargo before the capsid formation, which maintains the enzyme activity. In this work, such an approach reached a high load of cargo protein, an average of 20 molecules of CRISPR per particle (a significant load given the molecule size). The P22-CRISPR nanotool cleaved only the target sequence of dsDNA, so the cleavage activity and the specificity of CRISPR were not impaired inside the VLP. The system was also size specific in that the bigger molecules of DNA were not cleaved, because of the steric limitation from the pores’ size on the capsid [57].

Wang et al. [72] constructed the first nanocage designed for the biosynthesis of oligomeric biomolecules by using the *Pasteurella multocida* bifunctional glutathione full synthetases (GshFs). GshFs is an enzyme responsible for synthesizing glutathione (GSH), a protein responsible for detoxifying metabolites, including ROS and strong electrophiles in the liver. Given that P22 has previously shown predominant accumulation in the liver [119], it was used to encapsulate GshFs through a fusion to the N-terminus of a truncated SP (239–303), to be used for specific targeted delivery. Both SP-GshFs and CP were coexpressed in different plasmids, using two methods: simultaneous expression and late expression of CP. Unlike other enzymes reported before [66,70], the GshF had a higher catalytic activity after encapsulation through simultaneous expression than the free enzyme or through the CP late expression approach. This suggests that the extra time for maturation is not necessary for all enzymes and could depend on the complexity of the conformational change needed for the catalysis [72].

Although the total catalytic activity of the nanoreactor made of P22 VLP is higher than that of the free enzymes, this can be further improved by obtaining the best of the enzyme’s functionality. The individual enzyme activity reduction reported before may be related to factors such as (1) substrate diffusion limitation, which depends on the pores’ size and how the molecules are distributed inside the VLP; (2) enzyme confinement, which increases the interactions and limits the flexibility of enzymes once enzyme freedom is important to catalysis; and (3) changing the activation site, which can be affected by the crowding inside the VLP or by the genetic fusion to the SP as it affects the isoelectric point of the enzyme [66,70,71]. To overcome these problems, strategies were applied to take a precise control over the cargo concentration inside the VLP, which is hard to achieve by in vivo encapsulation. In one interesting example, Sharma et al. [66] performed an in vitro internalization of the alcohol dehydrogenase enzyme genetically fused to SP (SP-AdhD). To this end, CP and SPwt were obtained separately by heterologous expression by using different plasmids. The enzyme concentration was controlled by using different ratios of SP-AdhD and SPwt on the solution. The proteins assembled in regular and uniform VLPs, as in the in vivo technique. After the assembly, they used GuHCl to remove SPwt, maintaining only SP-AshD inside the VLP, given that it cannot pass through the 2.5 nm sized pores. The removal of SPwt decreased the molecular crowding, increasing the individual activity of each enzyme [66]. Additionally, previous studies have shown that the encapsulation in the VLP ensured a higher lifetime of the enzyme in vivo, by conferring resistance to high temperature and pH stability and to protease activity, which is a great quality for cancer treatment applications because there is an overexpression of extracellular matrix metalloproteinases (MMPs) in the tumor microenvironment, which can destroy the cargo protein. In addition, the VLP keeps the enzymes’ catalytic activity even in an oxygen atmosphere, which is important in the chemical and fuel industries [68,71].

In addition to enzymes, the VLP can be a nanoreactor using an inorganic catalyst, and its bioavailable but resistant shell makes it applicable for biological systems. Edwards et al. [95] encapsulated two catalysts with the xAEMA crosslinker, namely Eosin-Y, a photosensitizer, and cobaloxime, a catalyst that turns NAD+ into NADH or H_2_, to construct a bifurcated pathway catalyst system. Both were functionalized with N-chlorosuccinimide (NCS), which has a highly reactive N-Cl bond, used to crosslink with amine groups. The results showed that the VLP kept the catalysts in proximity, which enhanced the electron transfer needed for catalysis. The final system had a catalytic turnover higher than that of free catalysts, and it was independent of bulk concentration because the concentration inside the VLP remains constant. Furthermore, the pathway of reaction can be controlled; i.e., it is possible to induce the production of NADH or the production of H_2_ by varying the pH and the ratio between Eosin-Y and cobaloxime [95].

Synthesizing nanomaterials is a great challenge to material science owing to their high reactivity, which promotes aggregation and the loss of properties. The P22 VLP can act as a platform to synthesize these materials in an easy and controlled way. VLPs can trap inorganic cargoes by simple diffusion, exploiting their affinity for the cargo. Attaching auxiliary molecules on the SP and CP proteins, e.g., crosslinkers, can even more expand the variety of cargoes that can be encapsulated. Reichhardt et al. [99] showed that the inner SP apparently directed the nucleation of particles via an oxidative hydrolysis reaction, which can produce oxides from an ionic solution, where the CP layer constrained the growth of the particle and avoided interactions between other particles, limiting its size. Thus, they synthesized iron oxide nanoparticles (Fe_2_O_3_ NP) and yet improved the packaging by using a polyanionic peptide (ELEAE) fused to a truncated SP (239–303), which mimics the ferritin protein. It attracts the iron ions to the interior of the VLP, and it increases the homogeneity of NPs, resulting in spherical and highly monodisperse (41 ± 5 nm) NPs [99]. In another study, Zhao et al. [91] synthesized CdS nanoparticles by dispersing P22 with SP fused to a peptide of M13 phage with a specific binding to CdS, in a CdCl2 and thioacetamide (TA) solution. Without the P22 shell, the CdS nanocrystals assembled and formed nanoflowers that were 15–20 nm in diameter after 3 h of reaction. Inside the VLP, there were hexagonal monodisperse nanocrystals with an average size of 2.5 nm and polydisperse NPs with an average size of 5.8 nm, at 1 and 10 h of mixing, respectively. This indicates that the outer layer of VLP limited the size of the NPs, and the NPs’ growth also became more random with time, resulting in different NPs. Thus, it is possible to control the size of nanoparticles by controlling the time of the reaction [91].

Aside from particles, polymers were already synthesized inside the P22 VLP, as mentioned previously. For that, it is common to use a cysteine residue mutant of P22 without the SP to bind cysteine-reactive initiators. The sites have to be wisely chosen once the polymer access to the exterior surface can interfere in particle stability and promote interparticle connections, causing precipitation [78]). The K118C (exterior) and S39C (interior) are often used [64,78,93,94,95]. Uchida et al. [64] built a synthesis platform inside the ES morphology that controls and constrains polymer growth, making it a promising strategy for the synthesis of coordination polymers. The cysteine sites were modified with 5-iodoacetamido-1,10-phenanthroline (iodo-phen) to form a site for the initiation of coordination polymer growth and then alternatively mixed it with Ni, Co, or Fe compounds and 1,3-di-1,10-phenanthrolin-5-ylthiourea (Diphen) to form a metal–ligand coordination polymer inside the VLP. The majority of the polymer molecules were associated with the Ni-phen sites, and one-third of these sites were occupied by the coordination conjugate. This means that the interaction between the polymer and the P22 capsid is not relevant, because it is necessary at the initiation site of Ni-phen, which also allows the control of a number of polymer molecules by controlling the concentration of Ni-phen sites. In addition, the size and morphology of the VLP did not significantly change after the growth of the polymer. The final samples had a slightly broader mass distribution than that after the first addition of Ni and Diphen, which shows that the polymers are not homogeneous but instead have a finite molecular weight distribution. The xAEMA already mentioned was also synthesized inside the VLP without SP (ES, EX, or WB morphology). Through an atom-transfer radical polymerization (ATRP) with the cysteine sites modified with an ATRP initiator, monomers of AEMA and bisacrylamide formed a crosslinked polymer framework that can be used to attach a great variety of molecules, as it is a primary amine-rich molecule and can be functionalized with amine-affinity compounds [78,93,94,95]. To show that the amine sites were addressable, Lucon et al. [78] encapsulated fluorescent isothiocyanate (FITC) (on EX morphology), an amine-specific labeling agent with and without xAEMA. The xAEMA the FITC significantly increased loading, but the emission signal of fluorescence was less intense than the P22 without xAEMA, which suggests that the lack of space between polymer-bound fluorescein can disturb the fluorophore, as was seen for the MnPP CA.

## 10. Development of Supramolecular Structures

The VLPs can also be used as building blocks to form a three-dimensional active superlattice, which is a new research field that has been on the rise [73,87,90,120]. Uchida et al. [87] incorporated two repeats of a net negatively charged peptide with a sequence VAALEKE (E2 peptide) in the C-terminus of a coat protein (CP), which remains in the external surface of the VLP and causes repulsive interactions between the particles (Figure 6). After the assembly, the VLPs were mixed with positively charged amine-terminated generation 6 PAMAM dendrimer (G6), inducing an aggregation of the particles by electrostatic interactions between the positively charged G6 and the negatively charged P22 VLP. The attraction and repulsion ratio between the particles can be controlled by fine-tuning the ionic strength of the solution. A weak ionic strength induces the formation of amorphous aggregates, and a high ionic strength causes disassembly. However, at a threshold of ionic strength (I_T_ = 206 mM), the repulsion and attraction forces were equilibrated, producing an organized and well-spaced, crystalline, and face-centered cubic (FCC) structure. The optical microscopy of the P22-E2 superlattices revealed the presence of particulates with sizes in the range of 1–10 μm. One of the interesting uses for such a structure is as an efficient two-step catalyst, connecting VLPs with different enzymes that work together [87] through the encapsulation two enzymes (Ketoisovalerate decarboxylase (KivD) and alcohol dehydrogenase A (AdhA)) by genetic fusion to a truncated variant of an SP (141–303), at different VLPs. The assembly quality is independent of the cargo molecules inside the particle, and the maximum estimated enzyme concentration within the superlattices was shown to be almost three orders of magnitude higher than that in the free P22 VLP solution. Thus, the process of the two-step reaction is significantly faster than that of the noncondensed material, the free nanoreactors, or the free enzymes because of the higher substrate and catalyst concentrations, which could be beyond the solubility limit of some enzymes [87]. By using this electrostatic assembly as a template and attaching mutants of the Dec protein (S134C) to the VLP exterior, it is also possible to construct a stabler structure. The Dec proteins bind to each other through intermolecular disulfide bonds, forming a covalent Dec-Dec linker (Figure 6) [97]. In a high ionic strength medium, the G6 molecules are detached from the structure, but the FCC is maintained, with a negatively charged surface. The linker also increases the space between the VLPs, which improves the flow of substrate around the VLPs. The superlattice’s size makes it easy to be separated from the catalysis product, which can be recovered and recycled through centrifugation. Its activity can be maintained over multiple catalytic cycles.

## 11. Conclusions

The bacteriophage P22 has a remarkable research history, which began in the 1950s [20] and which has continued to develop and expand ever since. Its study was paramount in the advancement of our understanding of virus morphogenesis, especially studies on capsid maturation and protein subunits interactions [21,22,23,27,34,39,43,44,49,50]. This is in great part due to the fascinating structure that is the phage’s capsid, a deceptively simple icosahedral particle that can be formed solely through the interactions between coat protein subunits mediated by scaffold proteins. It is characterized by its relatively large size for a spherical phage particle and its capacity to undergo extensive structural transitions. It almost seems a natural progression that the research eventually turned to investigating the usage of this structure as a possible nanotool.

Throughout this review, the many virtues of the usage of the P22 capsid as a VLP were presented: from its efficient method of synthesis through heterologous expression [16] to the simple way that different variations of the VLP can be obtained [13,59]. Both properties enable the design of different methods through which cargo molecules can be internalized [13,25,57,63,64,65,78,82,84,85,93,95,121] or can decorate the particle [17,51,56,60,63,80,81]. This versatility has allowed the use of the P22 VLP in numerous applications, with an initial focus on its use in the targeted delivery of cargo molecules. Recently, the interest in its usage as a nanoreactor [64,69,78,89,91,92,93,94,96,98] has been growing thanks to the increasing number of studies reporting on methods for enzyme encapsulation [46,57,59,71,72,89,95,119]. Further propelling the usage of the P22 VLP for nanoreactor design is the growing tendency in the literature toward the development of macromolecular structures [29,66,88,90,121], thus creating VLP-based “nanofactories”.

However, another promising realm of application that has yet to be better explored is the P22 VLP’s immunogenic potential [83,117], particularly for the development of vaccine models [79,100,101]. It would be theoretically possible to create a multipotential immunogenic tool through the combination of therapeutic agent delivery [9,56,77,85,101] and antigen presentation [79,100,101,118], which could simultaneously control diseases such as cancer and induce long-lasting immunogenic protection. The study of the P22 VLP is a shining example of human ingenuity, and there is strong evidence that research has only just begun to show the true extension of this particle’s seemingly limitless potential.

## Figures and Tables

**Table 1:** The diverse types of cargo that have already been internalized using P22 VLP and the method used for encapsulation.

Cargo	Loading Method	Application
Fluorescent proteins mCherry [16,62,67] and GFP/EGFP [13,16,25,67,76,80]	Genetically fused to the truncated SP (141–303) protein [13,16,25,62,80], with a cysteine substitution (C140A) [81]; both proteins were fused with SP while being separated by oligomeric glycine spacers and encapsulated in vivo [67]	Study in vivo encapsulation of cargo [16,67]; probe the binding capability of glutamate modified P22 VLPs to hydroxyapatite [80]; used to probe cell internalization of VLP construct [62]; test the effects of cargo internalization on the VLP structure [25,82]; analyze cargo retention and release [13,76]
Influenza’s nucleuocapsid protein (NP), either whole or truncated [83]	Genetically fused to truncated SP (141–303) and encapsulated in vivo [83]	Test the potential for immunization in mice [83]
GALK-GLUK-CelB triple enzyme fusion synthetic metabolon [84]	Enzymes were genetically fused to truncated SP (141–303), starting with CelB, and interspaced with polyglycine peptides and encapsulated in vivo spacers [84]	Test the potential for in vivo encapsulation of enzymatic pathway and study the functionality of the construct [84]
M/M2 antigen fusion, consisting of respiratory syncytial virus (RSV) Matrix (M) and Matrix 2 (M2) [79]	RSV M/M2 protein C-terminus was fused to truncated SP (161–303) and encapsulated in vivo [79]	Test the usage of P22 VLP for viral antigens delivery and induction of immune response [79]
NuBCP-9 and KLAK peptides [85]	Peptide drugs were genetically fused to the N-terminus of P22 CP to ensure loading with a well-defined number of peptides [85]	Intracellular delivery of peptide drugs [85]
NADH oxidase (NOX), from the hyperthermophilic archeaon Pyrococcus furiosus [86]	Genetically fused to truncated SP (141–303) and encapsulated in vivo [86]	Test the potential of P22 VLP as a catalytically active construct that can kill and inhibit bacterial growth [86]
Cy7-maleimide [62]	Chemically conjugated to inserted cysteine (S39C) residue in VLP interior [62]	Used to probe cell internalization of VLP construct [62]
Clustered Regularly Interspaced Short Palindromic Repeats (CRISPR) and single-guide RNA [57]	Genetically fused to truncated SP and encapsulated in vivo [57]	Evaluate the P22 VLP potential for CRISPR transport and targeting of DNA for manipulation [57]
Ketoisovalerate decarboxylase (KivD) [87]	Directed self-assembly of capsid subunits with enzyme encapsulation and assembly of these VLP building blocks into three-dimensional arrays [87].	Test the enzymatic capabilities of a protein macromolecular framework construct [87]
Alcohol dehydrogenase D (AdhD) [29,66,82,87,88,89,90]	Genetically fused at the N-terminus of the truncated SP (141–303) [29,82,87,89,90]; coencapsulation with wild-type SP was performed at different stoichiometric ratios using an in vitro assembly approach [66]	Transport of targeted enzymes [89]; test the enzymatic capabilities of a protein macromolecular framework construct [87]; test the effects of cargo internalization on the VLP structure [29,66,82,87,88,89,90]); stoichiometry study of the enzyme [66]
Monomeric GCL and Homodimeric GshF [72]	Enzymes were genetically fused to the N-terminus of truncated SP (239–303) and encapsulated in vivo [72]	Elaboration of active nanoreactors capable of catalyzing the pathway for GSH biosynthesis [72]
Cadmium sulfide (CdS) nanocrystals [91,92]	Synthesized in the VLP interior, with growth centralized at the SP-fused peptide with binding specificity to CdS [91,92]	Synthesis of nanomaterials as visible light photocatalyst or bioimaging agent using the VLP as a nanoreactor [91]
SLTPLTTSHLRS peptide sequence [91,92]	Genetically fused to truncated SP (141–303) [91,92]	Confer CdS binding capability to P22 VLP and act as a nucleation site for the nanocrystal growth [91,92]
Manganese (III) protoporphyrin IX (MnPP) [93]	Conjugating each of the small molecules through an isothiocyanate moiety and covalent binding with crosslinked polymer (crosslinked aminoethyl methacrylate-AEMA) [93]	Evaluation of P22 VLP as a macromolecular vehicle for magnetic resonance imaging contrast agent [93]
Gadolinium (Gd(III)) functionalized with maleimido-monoamide (DOTA-mal/DTPA-mal) chelating agent complexes [63,94]	Covalent attachment with maleimido-monoamide (DOTA-mal/DTPA-mal) crosslinks at the cysteine mutation sites (K118C) in WB particle [63]/conjugating each of the small molecules through an isothiocyanate moiety and covalent binding with crosslinked polymer (crosslinked aminoethyl methacrylate-AEMA) [94]	Usage of Gd(III)-chelating agent-conjugated P22 viral capsids for in vivo magnetic resonance imaging (MRI) [63] Evaluation as a macromolecular magnetic resonance imaging contrast agent [94]
Eosin-Y isothiocyanate [95]	Conjugating each of the small molecules through an isothiocyanate moiety and covalent binding with crosslinked polymer (crosslinked aminoethyl methacrylate-AEMA) [95]	Photochemical production of NADH from NAD+ and H2 (and vice versa) under aqueous conditions [95]
Chloro(pyridine)cobaloxime (CoCl(dmgH)2Pyr) [95]	Conjugating each of the small molecules through an isothiocyanate moiety and covalent binding with crosslinked polymer (crosslinked aminoethyl methacrylate-AEMA) [95]	Photochemical production of NADH from NAD+ and H2 (and vice versa) under aqueous conditions [95]
Ziconotide (ω-MVIIA) analgesic peptide [56]	Genetically fused to truncated SP (238–303) [56]	Test the cell-penetrating peptide decorated P22 VLP potential for therapeutic agent delivery through the brain–blood barrier [56]
Cytochrome P450 from *Bacillus megaterium* (CYPBM3) [69,70,71]/glucose oxidase (GOx) functionalized CYPBM3 [96]	Genetically fused to truncated SP (141–303) and encapsulated in vivo, either simultaneously or prematurely expressed together with [70,71] and covalently conjugated with Gox using carbodiimide chemistry [96]; the particles were decorated with photosensitizer and targeting the portion on the surface for effective combinatorial treatment [69]	Study of P22 VLP potential for enzyme encapsulation, through differential expression and delivery [70]; used for the targeted delivery of therapeutic enzyme to carcinoma cells [71] Development of a multifunctional nanoparticle based on the integration of enzyme prodrug therapy and photodynamic therapy [69]; GOx conjugation enabled the transformation of EDCs by CYP in the presence of only glucose [96]
Maleimide-PEO2-biotin (MPB) or maleimide-C2-biotin [19]	Conjugated to inserted cysteine residues (V119C, K110C, and K118C) in ES and WB interior [19]	Probe-inserted mutations [19]
Fluorescein [19]/fluorescein isothiocyanate (FITC [78]/fluorescein-5-maleimide (F5M)) [9,12,81]	Conjugated to streptavidin (F-StAv) and bound to biotin terminals [19]; covalently bound to inserted cysteine residue (K118C) in WB particle [9]; covalently bound to AEMA polymer network synthesized in EX particle interior [78]	Probe-inserted mutations [19,81]; test the potential of P22 VLP-AEMA polymer construct for cargo loading [78]; used for cell imaging of internalized VLP construct [9]
Beta-glucosidase from the hyperthermophile Pyrococcus furiosus (CelB) [13,25,82,89,97]	Genetically fused to truncated SP (141–303) [13,25,82,89,97]	Study hierarchical assembly of a multilevel self-assembly system and the viability of the internalized tetrameric enzyme [89]; test the effects of cargo internalization on the VLP structure [25,82]; analyze cargo retention and release [13]; test the enzymatic capabilities of a protein macromolecular framework construct [97]
Catechol-Mal (*N*-(3,4-dihydroxyphenethyl)-3-maleimido-propa-namide) ligand [9]	Covalently bound to inserted cysteine residue (K118C) in WB particle interior [9]	Serve as conjugation site for bortezomib [9]
Bortezomib (BTZ) [9]	Covalently anchored to Catehol-Mal ligand in WB interior [9]	Test the potential of P22 WB targeted delivery against hepatocellular carcinoma (HCC) cells [9]
6-maleidocapyol hydrazine, a prodrug of doxorubicin (AlDox) [12]	Chemical conjugation by thiol-maleimide reaction into WB particle interior [12]	Develop a nanocarrier for the delivery of therapeutic agents for cancer treatment [12]
Asparginase [98]	Genetically inserted to SP [98]	Test the potential of P22 VLP for the delivery of therapeutic enzyme for the treatment of leukemia [98]
Polyanionic repeat of glutamic acid (ELEAE) [99]	Genetically fused to truncated SP (238–303) [99]	Used to enhance the formation of an iron oxide core (Fe_2_O_3_) [99]
Iron oxide nanoparticles (Fe_2_O_3_) [99]	Formed by oxidation of Fe^2+^ in the P22 VLP (PC, ES, SP_238_, and SP-ELEAE) interior [99]	Used to test the VLP potential as a nanoreactor for inorganic synthesis [99]
Iodo-phen (5-iodoacetamido-1,10-phenanthroline) [64]	Conjugated to inserted cysteine residues (K118C and S39C) to VLP interior [64]	Utilized to serve as site for functional molecule conjugation and initiation of polymer formation [64]
Coordination complex Nickel-phenanthroline (phen-Ni) [64]	Both Ni^2+^ and diphen (1,3-di-1,10-phenanthrolin-5-ylthiour-ea) linker molecules were coordinately bonded to initial phenanthroine-Ni conjugated to ES particle interior [64]	Demonstrate the coordination of ligand–metal network formation in the interior of the capsid [64]

**Table 2:** Different types of the functionalization of P22 VLPs.

External Insertion	Incorporation Method	Application
VLPs are used to create a core-shell structure with poly(4-vinylpyridine) polymers and polystyrene spheres [103]	Coassembly or template synthesis methods [103]	Molecular display [103]
Dec protein [60,61,62,90,97,102]	Electrostatically bound to the EX P22 VLP [60,61,62,102]; electrostatically bound Dec-Dec or Dec(4x)-Dps (DNA binding protein from starved cells) fusion proteins [90,102]; Dec-Dec electrostatically bound to the WB P22 VLP [97]	Serve as binding sites for nanogold beads and test Dec capability for molecular presentation [61]; used as a platform for the molecular display of antigens [62]; act as ditopic or tetratopic linkers molecule to promote interparticle interaction for the design of higher order assembly of particle structures/protein macromolecular framework [90,97,102]; test Dec potential to reinforce VLP and WB increase particle stability [60]
Nickel nitriloacetic acid (Ni-NTA) nanogold beads [61]	Bound to hexa-histidine tag inserted on the termini of Dec proteins [61]	Probe the accessibility of Dec protein for cargo presentation [61]
Self peptide (derived from CD47) [62]	Genetically fused to the C-terminus of the Dec protein and electrostatically bound to the EX P22 VLP [62]	Study the molecular presentation of Self peptide and diminish particle uptake by splenocytes [62]
Soluble region of murine CD40L [62]	Genetically fused to the C-terminus of the Dec protein and electrostatically bound to the EX P22 VLP [62]	Confer loaded EX P22 VLP affinity to B lymphocytes [62]
Diglutamate (E2) residue sequence [80]	Genetically inserted to P22 CP A-domain flexible loop (residues 180–185) [80]	Confer binding capability to hydroxyapatite [80]
RGD (Arginine-Glycine-Aspartic acid) peptide [51]	Inserted to T183 residue of the CP A-domain [51]	Evaluate tolerance of CP A-domain to manipulation [51]
Coiled coil peptides (E-coil, VAALEKE3/K-coil, VAALKEK3) [81,87,97]	Genetically inserted into polyhistidine (6xHis)-modified CP C- terminus [81,87,97]	Test the tolerance of P22 VLP C-terminus tolerance to extension and modification [81]; used to electrostatically interact with G6 to form a superlattices for the formation of macromolecular structures [87]; used to electrostatically generate an ordered template array for subsequent stabilization using Dec proteins for the design of protein macromolecular frameworks [97]
Fluorescein-5-maleimide (F5M) [81]	Bound to inserted cysteine residue at the end of CP C-terminus (431C) [81]	Test inserted mutation liability to chemical conjugation [81]
Gadolinium (Gd(III)) functionalized with maleimido-monoamide (DOTA-mal/DTPA-mal) chelating agent complexes [63]	Covalent attachment with maleimido-monoamide (DOTA-mal/DTPA-mal) crosslinks at the cysteine mutation sites (S133C) in WB particle exterior [63]	Usage of Gd(III)-chelating agent-conjugated P22 viral capsids for in vivo magnetic resonance imaging (MRI) [63]
SP94 (FSIIHTPILPL) ligand [9]	Chemically bound to SMCC (succinimidyl-4-(*N*-maleimidomethyl) cyclohexane-1-carboxylate) crosslinker conjugated to WB exterior surface [9]	Confer WB specificity to hepatocellular carcinoma (HCC) cells [9]
Gold nanoparticle [92]	Electrostatic interaction with histidine residue present in the P22 CP and exposed in the outer surface of the VLP [92]	Enhancing the photocatalytic of CdS [92]
Fluorescein isothiocyanate (FAM)-labeled HIV-Tat (YGRKKRRQRRR) cell-penetrating peptide [56]	Chemically attached to inserted cysteine residue (M338C) on P22 VLP exterior, postactivation with maleimidopropionic acid (MPA) [56]	Engineer a nanocontainer capable of delivering analgesic marine snail peptide ziconotide across the brain–blood barrier [56]
Norbornene (5-Norbornene-2-carboxylic acid) [76,77]	Chemically conjugated to lysine residues present on the exterior surface of P22 VLP [76,77]	Develop a method for P22 VLP programmed disassembly through ROMP reaction for the release of therapeutic peptides [76,77]
Sulfo-LC-SPDP (Sulfosuccinimidyl 6-(3′-(2-pyridyldithio)propionamido)hexanoate) cross linker [104]	Conjugated to mutant cysteine (M338C) residue inserted into CP I-domain [104]	Generate pyridyldithiol reactive groups on VLP exterior surface, subsequently reduced to thiols moieties, for ssDNA functionalization [104]
ssDNA with 12 nucleotides (5′-ACACACACACAC-3′) [104]	Chemically conjugated to PC surface through Sulfo-SMCC (4-(N-maleimidomethyl)-cyclohexane-1-carboxylate) crosslinker-mediated interaction [104]	Serve as linker between particle PC surface and ∆16-99Gag [104]
∆MA-CA-NC-SP2 (∆16-99Gag), a HIV Gag-derived protein [104]	Assembled in vitro in DNA-functionalized PC VLP surface [104]	Molecular presentation of HIV gag protein for structural analysis [104]
		
LPETG amino acid sequence on the C-terminus [17]	Genetically incorporated to CP C-terminus [17]	Used for sortase-mediated covalent binding of glycine-modified GFP and HAhead [17]
Green fluorescent protein (GFP) [17]; Biotin-SP-GFP [65]	Modified with glycine and enzymatically attached to inserted synthetic amino acid sequence exposed onto the VLP exterior [17]; GFP inserted in SP C-terminus while biotinylated N-terminus was conjugated to StAv-SP encapsulated inside PC particle [65]	Probe the potential to sortase-mediated covalent binding to VLP exterior [17]; used to bind to StAv-SP and anchor the SP-GFP N-terminus into the PC interior [65]
Head domain of influenza hemagglutinin protein (HAhead) [17,100]	Modified with glycine and enzymatically attached to inserted synthetic amino acid sequence exposed onto the VLP exterior [17]; SpyCatcher was inserted into HAhead [100]	Probe the potential to sortase-mediated covalent binding to VLP exterior [17]; display of antigen to induce immunity against influenza virus [100]
SpyTag/SpyCatcher [12,100]	SpyTag was genetically introduced to the C-terminus of the P22 capsid protein [12,100]	Molecular display of targeting ligands [12]; display of antigen to induce immunity against influenza virus [100]
Epidermal growth factor receptor affibody (EGFRaf) [12]	Genetically fused to SpyCatcher and bound to SpyTag-VLP [12]	Targeting ligand [12]
Human epidermal growth factor receptor2 affibody (HER2af) [12]	Genetically fused to SpyCatcher and bound to SpyTag-VLP [12]	Targeting ligand [12]
Polyethylene glycol/PEG-ESTAm [69,71,98]	Chemically conjugated to VLP surface through the activated succinimide moieties on PEG distal end [71]; PEG-containing estradiol derivative ESTAm was conjugated to VLP-exposed amine residue via Michael addition [69]; pegylated postassembly [98]	Test the potential of pegylation of VLP to mitigate immune response [71]; enable ROS generation for the elimination of MCF-7 cells [69]; kinetics and structural studies [98]
Folic Acid [71]	Decorated upon the surface of assembled P22 VLP through heterofunctionalized PEG [71]	Induce endocitosis of P22 VLP by cancer cells through folate-receptor-mediated endocytosis [71]
Protoporphyrin IX (PpIX) [69]	Coupled to exposed surface amines through carbodiimide chemistry [69]	Enable ROS generation for elimination of MCF-7 cells [69]
6-poly(amidoamine) dendrimer (G6) [87,90,97]	Electrostatically bound to VLP P22 decorated with E-coil peptide [87,90,97]	Used to electrostatically interact with E-coil peptide to form a superlattice for the formation of macromolecular structures [87,90]; used to electrostatically generate an ordered template array for subsequent stabilization using Dec proteins for the design of protein macromolecular frameworks [87]
Ovalbumin (OVA) B (ISQAVHAAHAEINEAGR) and T (SIINFEKL) epitopes [101]	Genetically inserted to the C-terminus of CP connected by a linker sequence (GGGGSGGGGS) [101]	Develop a P22-VLP-based antigen delivery platform for cancer immunotherapy [101]
Polyallylamine HCl (PAH) [88]	Assembled P22 VLP was incubated with 500 PAH chains per VLP in water at room temperature for polymer formation [88]	Evaluate the impact of functionalization with cationic polymers on the interactions of charged macromolecules and protein materials [88]
G6 polyamidoamine (PAMAM) [88]	Assembled P22 VLP was incubated with 400 G6 dendrimer per VLP in water at room temperature for polymer formation [88]	Evaluate the impact of functionalization with cationic polymers on the interactions of charged macromolecules and protein materials [88]

## Data Availability

Not applicable.

## Supplementary Materials

The following supporting information can be downloaded at https://www.mdpi.com/article/10.3390/v15020516/s1, Figure S1: Incorporation of VLPs (red) into T98G glioblastoma lineage; Figure S2: Incorporation of VLPs (red) into glioblastoma cells (green); Figure S3: Incorporation of VLPs (red) into neuronal cell cultures (green); Figure S4: Incorporation of VLPs (red) into astrocytes (green); Figure S5: In silico CPP predictions of all P22 proteins.
