# Supplementary material for: Bacteriophage P22 Capsid as a Pluripotent Nanotechnology Tool
Source: Viruses. 2023 Feb 13;15(2):516. doi: 10.3390/v15020516 (PMC9962691; doi:10.3390/v15020516)
Supplement: Supplementary file 1 [file viruses-15-00516-s001.zip › viruses-2167117-supplementary.pdf]

"Bacteriophage P22 capsid as a pluripotent nanotechnology tool" Supplementary Material

Methodology

1. Obtaining VLPs for incorporation tests in target cells

P22 VLPs were obtained as previously described by O'Neil and collaborators in 2011 [16], from heterologous expression in E. coli BL21DE3 cells. For this study, P22 VLPs containing mCherry fluorescent protein genetically fused to bacteriophage P22 scaffold protein were used.

2. Neuron culture

In order to evaluate the interaction of neuron cells and VLPs, the cerebral cortex of embryonic mice (E14) was used to obtain neuronal cells, following the protocol approved by the Animal Ethics Committee (CEUA), protocol n025/15. Dissection of the cerebral cortex was performed in a neurobasal medium (Gibco ™) supplemented with B27 (Invitrogem) [105]. The tissue was mechanically dissociated and the supernatant was collected for centrifugation. The centrifugation supernatant was discarded and the cells were resuspended in the neurobasal medium. Cells were harvested after mechanical tissue dissociation and resuspended in the neurobasal medium. For the experiment, 10 x 10^4 cells were plated per well, in a 24-well plate with coverslips treated with poly-L-lysine (Sigma). Obtaining the desired number of cells was performed by counting in a Neubauer chamber. The plated cells were incubated in the oven at 37°C with 5% CO2. After this period, 150μl of VLPs from a stock of 0.5 mg/ml was added to each well. The samples were incubated for two hours in an oven at 37°C and 5% CO2. Slides for confocal microscopy were prepared as described below.

3. Human astrocyte and glioblastoma (GBM02) culture

The human glioblastoma cell line GBM02 and the culture of human astrocytes were established at the Cellular Morphogenesis Laboratory (LMC), from samples of patients at the Clementino Fraga Filho University Hospital (HUCFF) in accordance with the ethics standards of the Ministry of Health, registered at the National Research Ethics Committee (CONEP) under registration number 2340, from 2001 [106,107]. The cultures were maintained in Dubelcco's Modified Eagle medium with F12 supplement (DMEM F12 - Gibco) with 10% Fetal Bovine Serum (FBS - Gibco) (MCS) in cell culture bottles. The cells were washed with phosphate-buffered saline (PBS) pH 7.4, 0.1 M. To detach the cells from the plate, 0.25% (Invitrogen) or ethylenediaminetetraacetic acid (EDTA) in PBS (PBS EDTA - Merk) at 37°C. The culture supernatant was collected and centrifuged. The cell pellet was resuspended in MCS. Cells were counted in a Neubauer chamber for plating. In 24-well culture plates with coverslips (Knittel Glass – Deckglase Cove slips 13mm) 5 x 10^4 cells were added per well. The plates with the cells were incubated for 24 hours in an oven at 37°C with 5% CO2 until they were used in the VLP incubation experiments as described below.

4. Tests for incorporation into target cells

In single cultures of glioblastoma cells (GBM02) or astrocytes, treatment with VLPs was performed 24 hours after plating. For neuron tests, the procedure was performed 1 week after plating. Before using the cells, all wells were washed twice with the respective culture medium.

Stocks for the experiment were prepared from the stock of purified P22 VLPs, as follows: VLP stock purified + medium without serum, for a final concentration of VLP 0.5 mg/ml in 150µL. From this stock, 150µL was added to each well. The control of the experiment was carried out with PBS + medium without serum, in the same proportion of the prepared stock of VLP. The plates were left incubating

in the oven at 37°C with 5% CO2, for the incubation time of each experiment, 30 minutes, 2 hours, or 24 hours.

For neuronal cell cultures, treatment with VLPs could only be performed one week after plating but followed the same incubation times described above. For cultures with glioblastoma and astrocytes, the VLP samples and the control (PBS) were diluted in DMEM. For neurons, the dilutions were made in the neurobasal medium.

After the incubation times for each treatment, the cells were fixed with 4% paraformaldehyde (Isofar) by incubation for 15 minutes. Cells were washed 3x with PBS and stored at 4°C to prepare for immunocytochemistry as described below.

5. Immunocytochemistry

For the analysis of the results, confocal fluorescence microscopy by immunocytochemical preparation was used. Samples were prepared by permeabilizing the cells with 0.2% Triton X-100 for 5 minutes. Subsequently, the cells were washed with PBS 1X 3 times and blocked with bovine serum albumin (BSA-AMRESCO) 5% for 30 minutes. Cells were incubated with primary antibody diluted in 1% BSA overnight at 4°C in a humid chamber. The primary antibodies used were anti-vimentin (1:200 dilution - Dako) and anti-MAP2 (1:1500 dilution - abcam).

After 12 hours, cells were washed 3 times with 1X PBS to remove unincorporated antibodies. Soon after, there was incubation with the secondary antibody alexa fluor 488 (1:400 dilution – Molecular Probes) diluted in 1% BSA for 2 hours in a humid chamber at room temperature. Thereafter, the cells were also washed with PBS 1X three times to remove excess unincorporated antibody. The labeling of

the cell nuclei with 4',6-diamidino-2-phenylindole dihydrochloride (DAPI – Sigma) was performed by adding it and incubating for 5 minutes and the cells were washed three times with 1X PBS after this incubation. Finally, the slide was mounted with the cover slips from each well of the plate, using fluoromount G (Southern Biotech) and for storage until microscopy. The slides were kept out of contact with light at a temperature of 4°C.

6. Confocal microscopy

The results of the incorporation tests were obtained through fluorescence confocal microscopy. With filters configured for mCherry, DAPI and ALEXA 488. Two microscope models were used or throughout the study. At CENABIO, ZEISS and LEICA microscopes were used, and at the multi-user microscopy center of the ICB UFRJ, LEICA microscopes were used.

After obtaining the images in microscopy, the softwares ZEN 2012 (ZEISS), LAS AF LITE (LEICA) and FIJI-ImageJ [108], were used for viewing and analyzing the images.

7. Cell penetrating peptides prediction

CPP predictions were made using the P22 proteins, whose function is described. The amino acid sequence of each protein was taken from the GenBank database (NCBI sequence: NC_002371.2) (https://www.ncbi.nlm.nih.gov/nuccore/51236723). Analyzes were performed using 4 CPP prediction software. First, we used CellPPD [109], where the complete protein sequence was added. The size of the resulting peptides was adjusted to 10 amino acids. The result of this prediction was all combinations of 10 amino acid peptides present in the protein. After this, the CPPs with the

highest CPP prediction score were chosen. The next step was to analyze each of the chosen peptides separately, as mentioned above. The other three software were used: SkiPCPP [110], CPPred [111] and MLCPP [112]. After closing all the proposed analyses, the results were compared and, based on that, the 3 peptides with the best scores were chosen.

Thinking about the functionality of these peptides, a cysteine was added to each sequence, always at one of the two ends. For this, all the previously mentioned analyzes were repeated, but now with the modified peptides. The final result, shown in supplementary figure 5, was obtained by comparing the best hits among all the evaluated peptides.

Results:

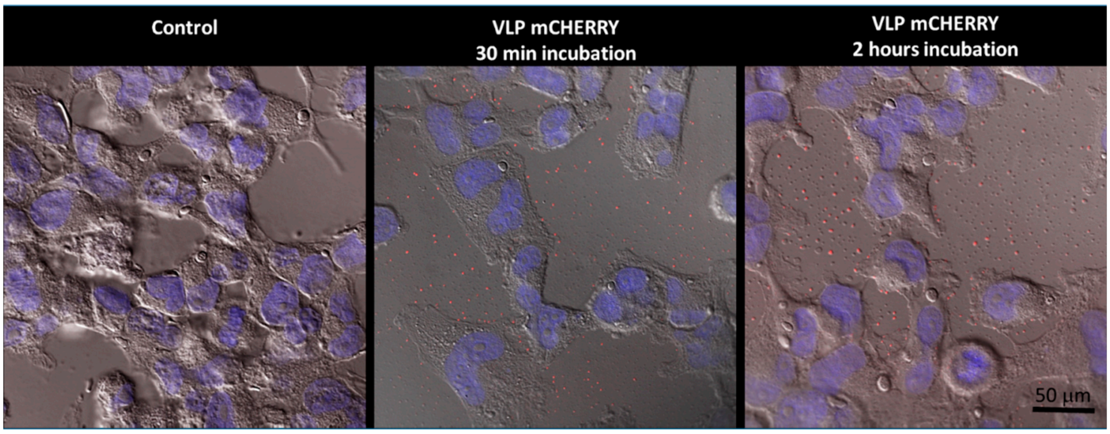

Figure S1: Incorporation of VLPs (red) into T98G glioblastoma lineage. Differential interference contrast (DIC) fluorescence microscopy images of VLP incorporation experiments in T98G cells with incubation for 30 minutes and 2 hours. DAPI-labeled cell nuclei and red fluorescent-labeled VLPs, mCherry. 50µm scale bar.

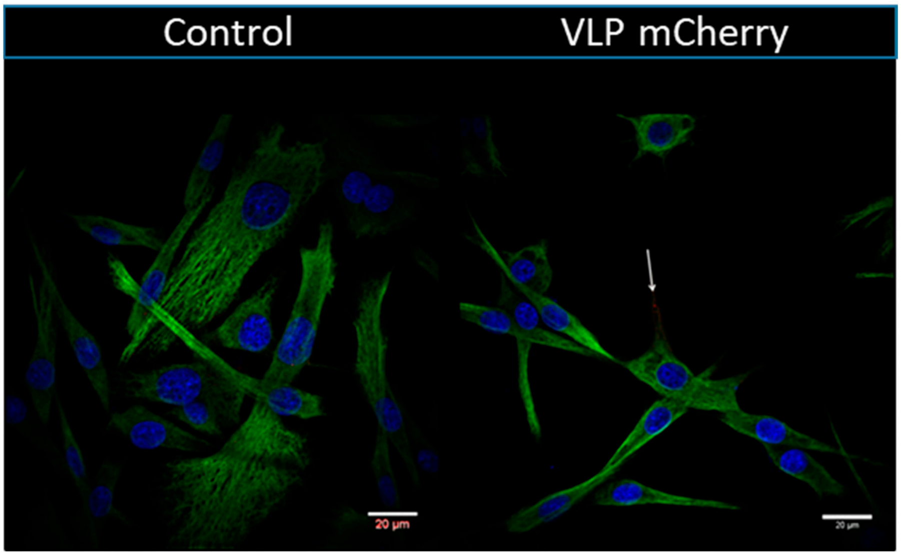

Figure S2: Incorporation of VLPs (red) into glioblastoma cells (green). Confocal fluorescence microscopy images show the overlap of the 3 fluorescence fields, cells labeled in green (ALEXA 488), cell nuclei labeled in blue (DAPI) and VLPs labeled in red (mCherry). Incubation of cells with VLPs for 2 hours. This time was chosen based on previous analyzes. The arrow indicates the location of the presence of the VLPs. 20μm scale bar. N of experiments equal to 3.

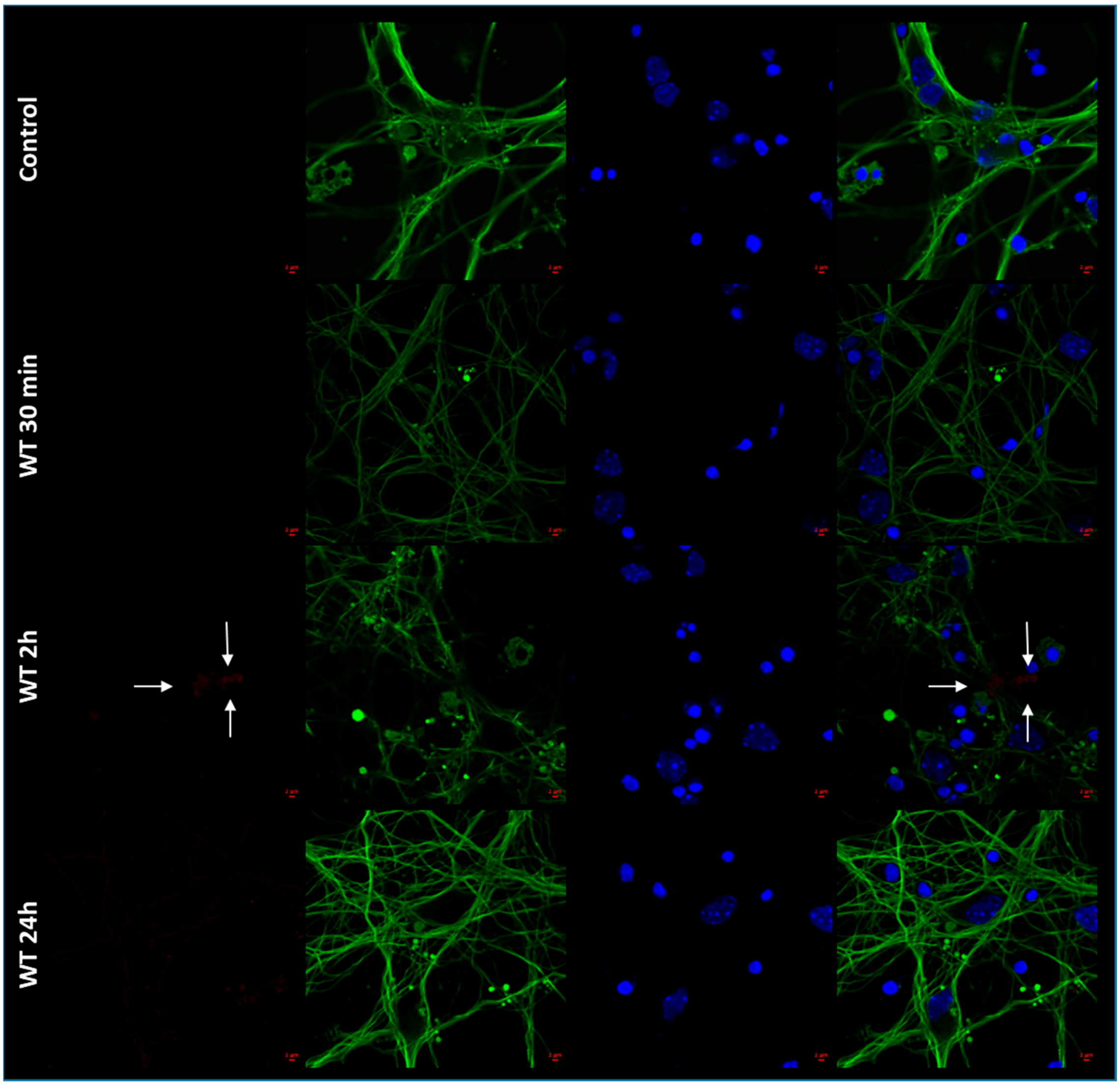

Figure S3: Incorporation of VLPs (red) into neuronal cell cultures (green). Fluorescence confocal microscopy images. The first column corresponds to the microscopy field for obtaining red fluorescence (mCherry). The second column corresponds to the field for obtaining green fluorescence (ALEXA 488). The third column corresponds to the field for obtaining blue fluorescence (DAPI). The last column corresponds to the overlap of all previous fields. Arrows indicate the location of the red tag (VLPs-mCherry). 2μm scale bar.

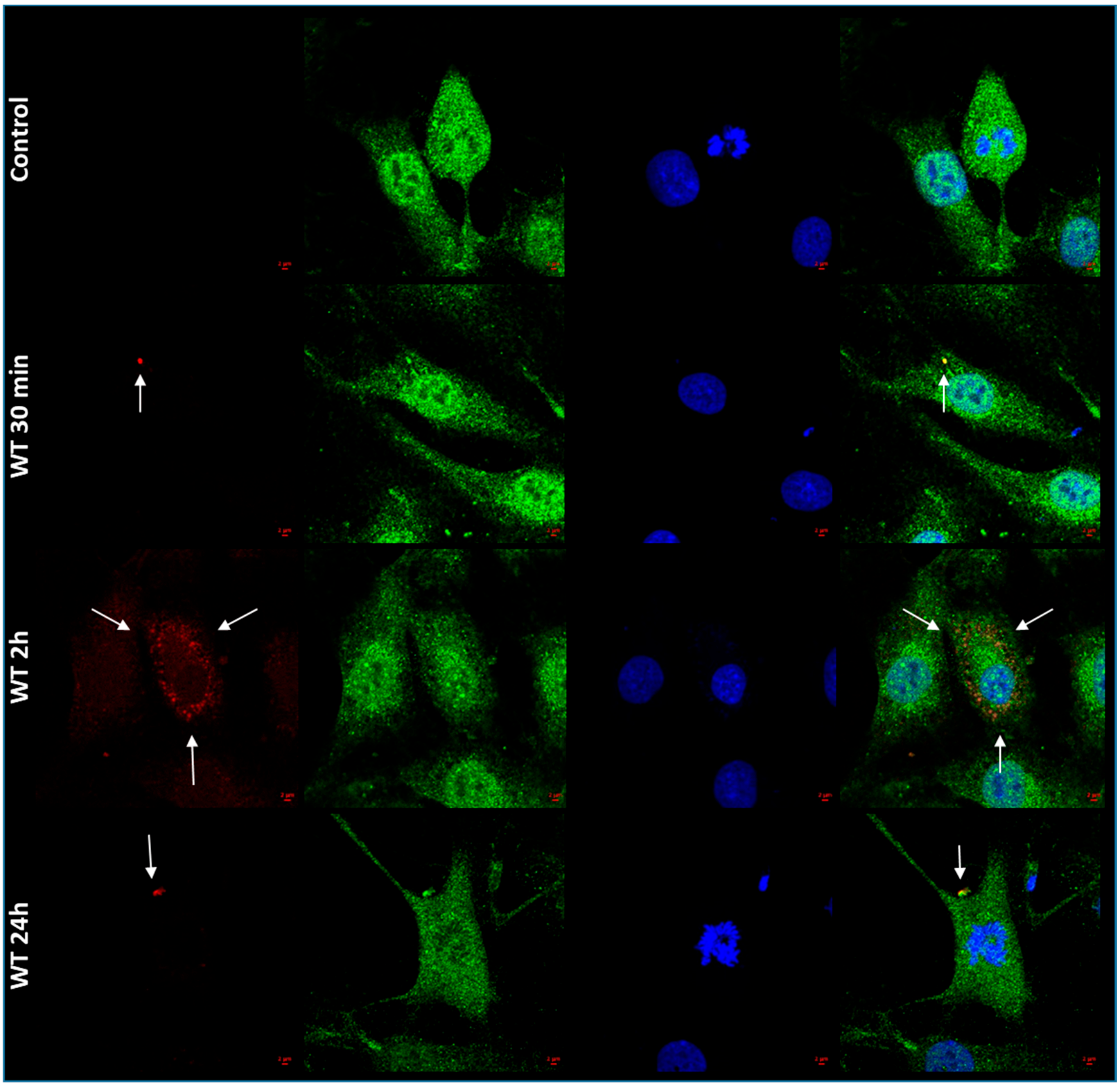

Figure S4: Incorporation of VLPs (red) into astrocytes (green). Fluorescence confocal microscopy images. The first column corresponds to the microscopy field for obtaining red fluorescence (mCherry). The second column corresponds to the field for obtaining green fluorescence (ALEXA 488). The third column corresponds to the field for obtaining blue fluorescence (DAPI). The last column corresponds to the overlap of all previous fields. Arrows indicate the location of the red tag (VLPs-mCherry). 2μm scale bar. N of experiments equal to 3

A:

Sequence	Software	Prediction	Prediction score
CTRRHERRRK	CellPPD	CPP	0.66
	SkipCPP	CPP	0.768
	CPPred	CPP	0.737
	MLCPP	CPP	0.892

B:

Sequence	Software	Prediction	Prediction score
CTRRHERRRK	CellPPD	CPP	0.84
	SkipCPP	CPP	0.713
	CPPred	CPP	0.870
	MLCPP	CPP	0.954

C:

Sequence	Software	Prediction	Prediction score
RRRIERKRQC	CellPPD	CPP	0.75
	SkipCPP	CPP	0,786
	CPPred	CPP	0.796
	MLCPP	CPP	0.943

D:

Sequence	Software	Prediction	Prediction score
CTRRHERRRK	CellPPD	CPP	0.98
	SkipCPP	CPP	N/A
	CPPred	CPP	0.915
	MLCPP	0.992	0,892

Figure S5: In silico CPP predictions of all P22 proteins. Result of predictions comparing 4 different programs. A: portal protein; B: superinfection protein; C: Scaffold protein; D: experiment control, Tat HIV protein. Peptides were predicted with the original sequence, changing one of the 10 amino acids by a cysteine, either at the C-terminus or N-terminus. Thus, cysteine would be used as a site for binding with cysteine regions of the P22 capsid protein.
